# Ion channel-gated covalent organic framework membrane for sustainable lithium–sulfur batteries

**DOI:** 10.1093/nsr/nwaf193

**Published:** 2025-05-16

**Authors:** Zhongping Li, Jae-Seung Kim, Hyunseok Moon, Kyeong-Seok Oh, Yuxin Hou, Sodam Park, Kun Ryu, Changqing Li, Jeong-Min Seo, Xiaoming Liu, Jong-Beom Baek, Dong-Hwa Seo, Sang-Young Lee

**Affiliations:** Key Laboratory of Automobile Materials of MOE and School of Materials Science and Engineering, Jilin University, Changchun 130012, China; Department of Chemical and Biomolecular Engineering, Yonsei University, Seoul 03722, Republic of Korea; Department of Materials Science and Engineering, Korea Advanced Institute of Science and Technology (KAIST), Daejeon 34141, Republic of Korea; Department of Chemical and Biomolecular Engineering, Yonsei University, Seoul 03722, Republic of Korea; Department of Chemical and Biomolecular Engineering, Yonsei University, Seoul 03722, Republic of Korea; College of Chemistry, Jilin University, Changchun 130012, China; School of Energy and Chemical Engineering, Ulsan National Institute of Science and Technology (UNIST), Ulsan 44919, Republic of Korea; Pritzker School of Molecular Engineering, The University of Chicago, Chicago, IL 60637, USA; School of Energy and Chemical Engineering, Ulsan National Institute of Science and Technology (UNIST), Ulsan 44919, Republic of Korea; College of Chemistry, Jilin University, Changchun 130012, China; College of Chemistry, Jilin University, Changchun 130012, China; School of Energy and Chemical Engineering, Ulsan National Institute of Science and Technology (UNIST), Ulsan 44919, Republic of Korea; Department of Materials Science and Engineering, Korea Advanced Institute of Science and Technology (KAIST), Daejeon 34141, Republic of Korea; Department of Chemical and Biomolecular Engineering, Yonsei University, Seoul 03722, Republic of Korea; Department of Battery Engineering, Yonsei University, Seoul 03722, Republic of Korea

**Keywords:** anionic covalent organic frameworks, ion channel, ionic sieve membrane, charge delocalization, dissociation of Li^+^, Li–S batteries

## Abstract

Lithium–sulfur (Li–S) batteries hold promise as a compelling alternative to current state-of-the-art Li-ion batteries due to their high theoretical capacity, low cost and the natural abundance of sulfur. However, the practical realization of Li–S batteries has been plagued by the longstanding trade-off issue between polysulfide shuttle suppression and Li⁺ transport. Here, we report an ion channel-gated covalent organic framework (COF) as an ionic diode membrane strategy to address this conflicting requirement. By tuning the chemical structure of tethered anions, the resulting COF features 1D anionic channels with optimized charge delocalization and pore size. The bulky anions enhance Li⁺ dissociation and conduction while effectively repelling polysulfides dissolved from S cathodes. Additionally, the COF ionic diode mitigates self-discharge and inhibits parasitic reactions. Consequently, Li–S cells assembled with the COF ionic diode improve charge/discharge capacities and cycle life under constrained operating conditions.

## Teaser

This work reports ion channel-gated, charge-delocalized anionic COFs as electrostatic permselective membranes to address the trade-off between selectivity (shuttle prevention) and permeability (Li^+^ conduction) in LiS batteries.

## INTRODUCTION

The ever-increasing demand for advanced batteries with high energy density and a long cycle life has inspired the relentless pursuit of new electrochemical systems and electrode materials. However, there are challenges associated with augmenting the charge storage capability of intercalation-based transition metal oxide cathodes, which are prevalently used in current state-of-the-art Li-ion batteries, as well as enhancing their performance to meet the evolving requirements of the competitive markets [1–3]. Consequently, this has prompted extensive research into alternative cathode materials. Owing to its high theoretical capacity (1672 mAh g^−1^), low cost, environmental friendliness and natural abundance, sulfur has been proposed as a promising cathode material [4,5]_._

Despite the notable benefits of Li–S batteries [6,7], several challenges, particularly the ‘shuttle effect’ of Li polysulfides (PSs), have hindered their practical application. Accordingly, numerous research efforts have been devoted to resolving the problem of the PSs shuttle effect [8–11], including the modifications of sulfur cathodes [12–17], electrolyte engineering [18,19] and the protection of Li-metal anodes [20,21]. However, these methods often fail to effectively suppress diffusion of PSs, resulting in poor capacity retention. Moreover, the increased proportion of inactive cell components and the complex processability hinder their practical viability. In contrast to the aforementioned approaches, membranes incorporating functional layers, such as carbon [22–24], inorganic [25–27] and hybrids [28,29], have attracted considerable attention due to their high selectivity for PSs, resulting in improved cyclability. However, these approaches often lead to a compromise between inhibiting the PSs shuttle effect and facilitating Li^+^ conduction. This trade-off becomes particularly pronounced under constrained electrolyte conditions (e.g. low electrolyte-to-sulfur (E/S) ratios of <5 μL mg_sulfur_^–1^) and in high-capacity cells (>5 mAh cm^–2^). Therefore, there is an urgent need to develop a new membrane concept that can effectively suppress the PSs shuttle effect without impairing the Li^+^ transport phenomena. Among the many membrane materials reported to date, covalent organic frameworks (COFs) [30–36] have recently emerged as a promising membrane material owing to their directional porous structure, structural stability and chemical tunability [37–50,51]. Additionally, from a battery perspective, the functional and aligned 1D pore channels of COFs are expected to enable chemical selectivity and facilitate charge transport [52–55].

**Figure 1.**
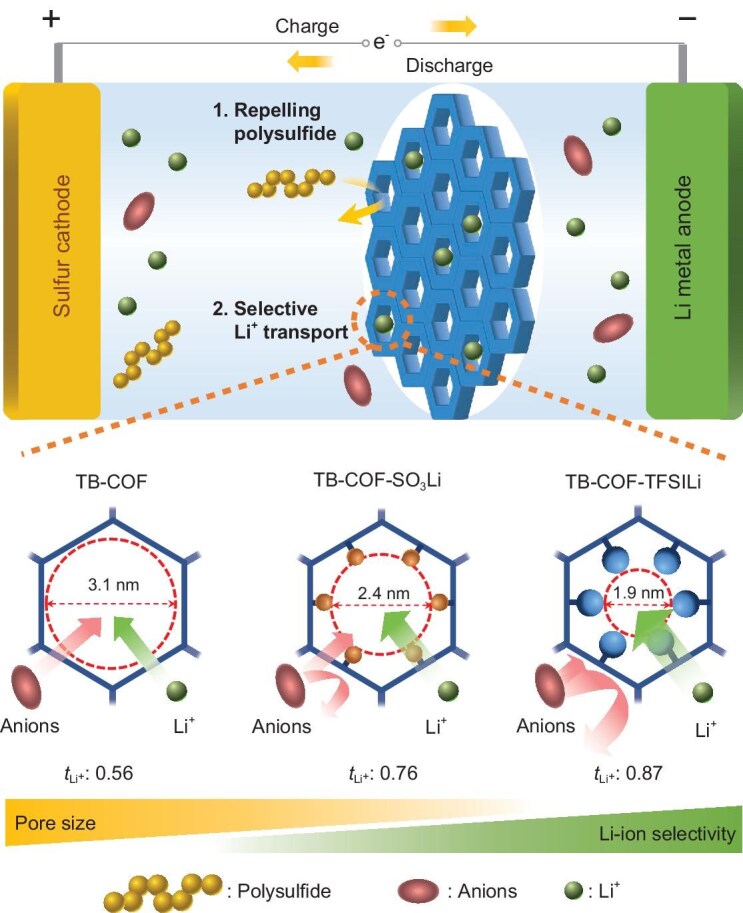
Schematic illustration of the fabrication of anionic COFs as an ionic sieve membrane for Li–S batteries.

In this study, an ion channel-gated COF with charge-delocalized, narrowed pores is introduced as a class of ionic diode membranes to address the persistent trade-off challenge between the PSs shuttle prevention (selectivity) and Li^+^ conduction (permeability) (Fig. 1). The charge delocalization and pore size of the anionic channels were regulated by varying the chemical structure of tethered anions. Particularly, imine-containing COFs were synthesized with different anions (sulfonate (SO_3−_) and bis(trifluoromethane)sulfonimide (TFSI_−_)). Whereas previous studies have primarily focused on attaching functional groups to the COF skeletons [50,56–59], we aimed to regulate the pore size and charge density distribution of anionic COF channels by incorporating ionic groups inside the COFs. The incorporation of a bulky anion (TFSI^−^) into the COF channels facilitated charge delocalization and pore-size reduction, thereby effectively repelling polysulfides through a synergistic combination of electrostatic interactions and steric hindrance. Furthermore, the delocalized negative charge distribution suppressed anion transport while promoting Li⁺ dissociation, enabling highly selective (transference number of Li⁺ *t*_Li⁺_ = 0.87) and efficient Li⁺ conduction through the COF channels.

## RESULTS AND DISCUSSION

### Structure and characterization of ion channel-gated COFs

**Figure 2.**
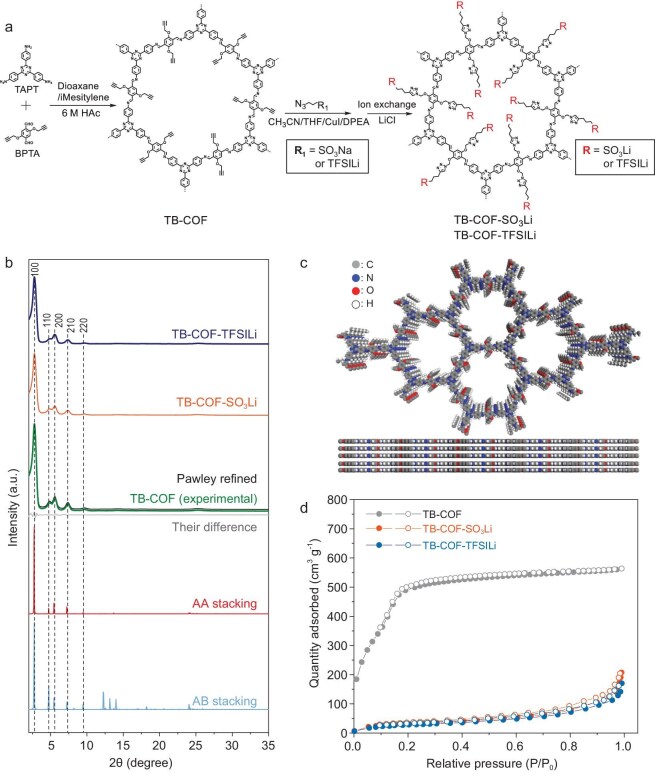
Structure and characterization of the TB–COF, TB–COF–SO_3_Li and TB–COF–TFSILi. (a) Synthetic scheme of COFs. (b) Experimental and theoretical PXRD patterns. (c) Theoretical unit cells of the TB–COF. (d) Nitrogen sorption isotherms measured at 77 K (solid: adsorption; hollow: desorption).

In this study, an imine-based TB–COF was first synthesized by mixing 2,5-bis(prop-2-yn-1-yloxy) terephthalaldehyde (BPTA) and 2,4,6-tris(4-aminophenyl)-1,3,5-triazine (TAPT) in a solution of dioxane and mesitylene with 6 M acetic acid used as a catalyst for 3 days at 120°C. Thereafter, the highly charged TB–COF–SO_3_Li and TB–COF–TFSILi were synthesized by post-functionalization of the channel walls of the pre-synthesized TB–COF (for details, see Fig. 2a and experimental section, Notes S1–S3). The morphologies of the obtained TB–COF, TB–COF–SO_3_Li and TB–COF–TFSILi were characterized (Figs S1 and S2). Overall, no significant difference in the morphologies of these COFs was observed, indicating that the post-functionalization of the TB–COF did not affect the morphologies of the resulting modified COFs. Signals corresponding to the imine bond and tri-bond were observed in the Fourier transform infrared (FT–IR) spectra of TB–COF at 1576 and 2129 cm^−1^, respectively (Fig. S3, green). Additionally, a strong peak was observed in the ^13^C magic angle spin solid-state nuclear magnetic resonance (NMR) of TB–COF at 160.4 ppm, corresponding to the carbon atoms within the imine linkage (Fig. S4a). Furthermore, notable carbon peaks of the tri-bond were observed at 72.4 and 78.3 ppm. After the post-synthesis, the tri-bond signals within the FT–IR spectra of TB–COF–SO_3_Li (Fig. S3, orange) and TB–COF–TFSILi (Fig. S3, blue) were dispersed. In addition, the carbon signal of the tri-bond disappeared and new peaks from triazine units emerged at 143.1 ppm (Fig. S4b). Further, a strong peak was observed at −71.5 ppm, which corresponded to the fluorine atoms of TB–COF–TFSILi (Fig. S4c). The surface chemistry of TB–COFs was analysed by using X-ray photoelectron spectroscopy (XPS), which revealed different element distributions. The peaks of carbon (C 1s), nitrogen (N 1s), oxygen (O 1s), sulfur (S 2p), and lithium (Li 1s) were observed in the XPS profiles of TB–COF, TB–COF–SO_3_Li and TB–COF–TFSILi (Fig. S5a–c). Particularly, the TB–COF–TFSILi showed a strong peak of F 1s at 668.0 eV, indicating the presence of TFSI^−^ as a tethered anion (Fig. S5c). Meanwhile, a characteristic peak of Cl 1s at 199 eV was not detected at both the TB–COF–SO_3_Li and TB–COF–TFSILi, exhibiting the successful removal of LiCl during the ion-exchange step.

The crystalline properties of COFs were investigated by using powder X-ray diffraction (PXRD) measurements. The PXRD pattern of TB–COF exhibited distinct diffraction peaks at 2.78° (100), 4.90° (110), 5.58° (200), 7.38° (210), 9.72° (220) and 25.24° (001), confirming its high crystallinity (Fig. 2b, green). After Pawley refinement, the observed PXRD pattern matched well with the simulated patterns from the AA-staggered stacking model (Fig. 2b, red). The optimized cell parameters of TB–COF showed good agreement factors (*R*_p_ = 2.77%, *R*_wp_ = 3.78%, *a* = *b* = 37.3302 Å, *c* = 3.6985 Å, *α* = *β* = 90° and *γ* = 120°, Fig. 2c). The TB–COF–SO_3_Li (Fig. 2b, orange) and TB–COF–TFSILi (Fig. 2b, blue) also maintained a high crystallinity. The specific surface area was determined by using the Brunauer–Emmett–Teller method (Fig. 2d). TB–COF exhibited a high surface area of 1728 m^2^ g^−1^ [1]. In comparison, TB–COF–SO_3_Li and TB–COF–TFSILi revealed lower surface areas of 81 and 72 m^2^ g^−1^ [1], respectively. In addition, the pore-size distribution of the COFs was analysed by using N_2_ adsorption–desorption isotherms (Fig. S6). The average pore-size values of the TB–COF, TB–COF–SO_3_Li and TB–COF–TFSILi were estimated to be 3.1, 2.4 and 1.9 nm, respectively. We confirmed that the average pore sizes of these COFs are consistent with the theoretical density functional theory (DFT) values obtained from the calculation of six-layer stacked COFs. It was observed that the cross-sectional area of TB–COF–TFSILi (240.0 Å²) was lower than those of TB–COF–SO_3_Li (433.0 Å²) and TB–COF (744.0 Å²) (Table S1). As the volumetric flow rate of molecules is directly proportional to the cross-sectional area according to Darcy's law [60], the reduced channel dimensions in TB–COF–TFSILi effectively suppress the transport of bulky PSs and TFSI⁻ anions, thereby enhancing the ion-sieving effect. These results confirm that TB–COF–TFSILi was successfully synthesized via wall-channel functionalization, demonstrating its ability to selectively regulate ion transport while mitigating polysulfide crossover.

### Effect of tethered anions in COFs on Li^+^ dissociation and interaction with PSs

**Figure 3.**
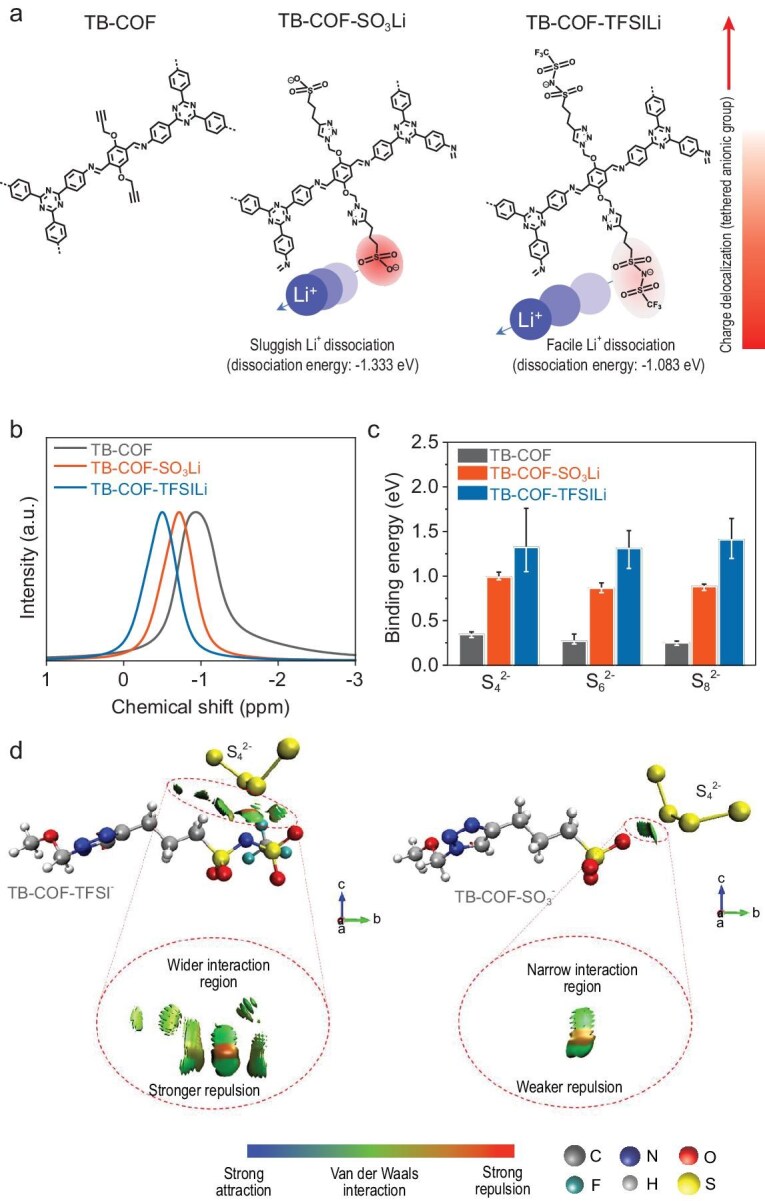
Effect of tethered anions in the COFs on Li^+^ dissociation and interaction with PSs. (a) Chemical structures of the weaker ion correlations expected in systems employing larger ions with greater charge delocalization. The degree of correlation is shown by the scale bar on the right. (b) Solid-state ^7^Li NMR spectra of COFs. (c) Gibbs free energy of binding between COFs and S*_n_*^2–^. The main color bar represents the median binding energy value, calculated from 20 structures of each functional motif in COFs and each PSs composition. Additionally, the gray error bars indicate the range of binding energy values. (d) Non-covalent interactions within 3D molecular structures of the functional group in the TB–COF and PSs. TB–COF–TFSI^–^–S_4_^2–^ (left) and TB–COF–SO_3_^–^–S_4_^2–^ (right).

DFT calculations were performed to evaluate the dissociation energy between Li^+^ and the –SO_3_– and –TFSI–COF motifs. The dissociation energy of SO_3_Li (−1.333 eV) was higher than that of TFSI–Li (−1.083 eV) (Fig. 3a and Fig. S7), indicating that Li⁺ dissociates more readily from TFSI⁻ than from SO_3_⁻. To further investigate the charge distribution, molecular electrostatic potential (MESP) plots of the functional groups were analysed (Fig. S8). Delocalized negative charge sites were observed in the patterns of TFSI^−^, whereas the patterns of –SO_3_^−^ presented a high charge density and negative potential. Consequently, the dissociation of Li^+^ from SO_3_^−^ is expected to be more challenging. Next, the local chemical environment of Li^+^ in the COFs was investigated by using ^7^Li NMR spectroscopy (Fig. 3b). The ^7^Li NMR spectra revealed that TB–COF–TFSILi demonstrated a narrower peak width compared with TB–COF and TB–COF–SO_3_Li, indicating the enhanced mobility of free Li^+^. Additionally, there was a downshift in the ^7^Li NMR peak, suggesting the effective dissociation of Li salts [53,54].

Subsequently, the interactions between the functional groups of COFs and PS anions (S*_n_*^2−^) were investigated by using DFT calculations (Fig. 3c and Fig. S9). The results revealed that TB–COF–TFSI^−^ exhibited a pronounced tendency for repulsive interactions with PS compared with TB–COF–SO_3_^−^. We further employed non-covalent interaction (NCI) calculations to obtain the 3D mapping of these interactions (Fig. 3d). The NCI calculations intuitively revealed the attractive and repulsive forces between molecules. Remarkably, TB–COF–TFSI^−^ displayed wider interaction regions and stronger repulsive interactions with PS, suggesting that the presence of a larger, bulky anionic functional group facilitated enhanced repulsion against PS. In particular, the delocalized charge density distribution and the steric hindrance of the bulky TFSI^−^ anion effectively repelled the PS anions.

### Transport phenomena of ion-channel-gated COF

**Figure 4.**
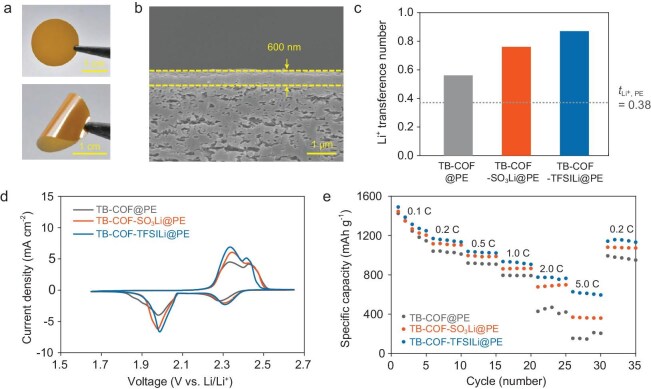
Ion-transport phenomena of the COF membranes. (a) Optical images of the TB–COF–TFSILi@PE. (b) Cross-sectional SEM image of the TB–COF–TFSILi@PE. (c) Li^+^ transference number of the pristine PE and various COF@PEs. (d) Comparison of the CV curves of Li–S cells with the COF@PEs. (e) Rate performance of the COF@PEs (sulfur loading = 1.0 mg cm^‒2^; E/S ratio = 10 μL mg_sulfur_^‒1^).

The as-synthesized COF was coated on a polyethylene (PE) separator to obtain COF@PE, which was characterized by a yellow color on the COF-coated side (Fig. 4a, top). The COF@PE demonstrated superior flexibility, with no noticeable delamination, even after bending (Fig. 4a, bottom). Scanning electron microscopy (SEM) images of the COF@PE revealed that the PE separator was uniformly covered by the compactly packed COF layer (Fig. S10). The thickness of the COF layer was ∼600 nm (Fig. 4b). To verify the electrolyte wettability of the TB–COF–TFSILi@PE, contact-angle measurement was conducted by dropping a liquid electrolyte (1 M LiTFSI in 1,3-dioxolane (DOL) / 1,2-dimethoxyethane (DME) + 2 wt% LiNO_3_) on the pristine PE and TB–COF–TFSILi@PE (Fig. S11). The TB–COF–TFSILi@PE showed a smaller contact angle (19.0°) compared with the pristine PE (25.8°), indicating its improved electrolyte wettability. This improvement can be attributed to the affinity of the negatively charged framework of TB–COF–TFSILi towards the polar electrolyte.

To demonstrate the effect of anionic charge delocalization, the ionic conductivities of the COF pellets as well as those of the COF@PE were analysed after impregnation with a liquid electrolyte (1 M LiTFSI in DOL/DME = 1/1 (v/v)). The ionic conductivities of the COF pellets at room temperature tended to increase in the following order: TB–COF (1.56 × 10^−4^ S cm^‒1^), TB–COF–SO_3_Li (3.68 × 10^−4^ S cm^‒1^) and TB–COF–TFSILi (6.16 × 10^−4^ S cm^‒1^) (Fig. S12). This result indicates that the delocalized negative charge of the TB–COF–TFSILi can contribute to the enhancement of ion conduction, which is consistent with the MESP plots obtained from the DFT calculation (Fig. S8) and ^7^Li NMR spectra (Fig. 3b). A similar trend was observed in the ionic conductivity of the COF@PE. The TB–COF–TFSILi@PE showed higher ionic conductivity (3.77 × 10^−4^ S cm^−1^) than the TB–COF–SO_3_Li@PE (3.61 × 10^−4^ S cm^−1^) and the TB–COF@PE (3.54 × 10^−4^ S cm^−1^) (Fig. S13). Compared with the result obtained for the COF pellets, this slight difference in the ionic conductivity could be attributed to the thin COF layer (∼ 600 nm) deposited on the PE separator (∼ 20 μm).

The effect of COF@PE on the Li^+^ transference number was determined by using the Bruce–Vincent method. The results revealed a higher Li^+^ transference number of TB–COF–TFSILi (0.87) compared with those of pristine PE (0.38), TB–COF (0.56) and TB–COF–SO_3_Li (0.76) (Fig. 4c, Fig. S14 and Table S2), which can be attributed to the high ionic dissociation and smaller pore size [16,61]. This higher Li^+^ transference number can beneficially contribute to increasing the Li^+^ conductivity (= ionic conductivity × *t*_Li^+^_). The Li^+^ conductivities of the TB–COF–TFSILi@PE, TB–COF–SO_3_Li@PE, TB–COF@PE and pristine PE were estimated to be 3.28 × 10^‒4^, 2. 81 × 10^‒4^, 2.00 × 10^‒4^ and 2.70 × 10^‒4^ S cm^‒1^, respectively. Consequently, the difference between these membranes was more pronounced for the Li^+^ conductivity compared with the ionic conductivity shown in Fig. S13.

To investigate the effect of the COF@PEs on the redox reactions of Li–S cells, cyclic voltammetry (CV) experiments were conducted in the potential range of 1.65–2.65 V vs. Li/Li^+^ at a scan rate of 0.1 mV s^−1^ (Fig. 4d). The positions of two oxidation and two reduction peaks, corresponding to the electrochemical conversion reactions [55] between sulfur and Li_2_S_2_/Li_2_S, were analysed. For the TB–COF–SO_3_Li, the oxidation peaks appeared at 2.35 and 2.42 V, which were lower than those observed for the TB–COF@PE and TB–COF–SO₃Li@PE, indicating the improved conversion reaction kinetics of polysulfides, potentially due to the catalytic effect [62] of the TB–COF–SO_3_Li. Additionally, the height gaps of the main peaks slightly increased with the lager anion size, indicating the improved Li–S battery reversibility of the TB–COF–TFSILi@PE compared with the TB–COF@PE and TB–COF–SO_3_Li@PE. Next, the rate capability of the samples was compared to investigate their Li^+^ transport effects (Fig. 4e and Fig. S15). The Li–S cell with the TB–COF–TFSILi@PE showed high discharge capacities of 1247, 1169, 1038, 934, 776 and 627 mAh g^–1^ at C-rates of 0.1, 0.2, 0.5, 1, 2 and 5 C, respectively, and the capacity was restored to 1157 mA h g^−1^ when the C-rate was returned to 0.2 C, which were comparable to those of previous works [49,56–58]. In contrast, the Li–S cells with the TB–COF@PE and TB–COF–SO_3_Li@PE exhibited notable capacity decay at high rates. These results indicated that the TB–COF–TFSILi@PE contributed to facilitating the charge/discharge reaction kinetics in Li–S cells.

The dependence of the scan rate on the current response was utilized to calculate the Li^+^ diffusion coefficients (*D*_Li*^+^*_) by using the Randles–Sevcik equation [63]:


\begin{eqnarray*}
{I}_{\mathrm{p}} = 2.69 \times {10}^5{n}^{1.5}A{D}_{{\mathrm{L}}{{\mathrm{i}}}^ + }^{0.5}{C}_{{\mathrm{L}}{{\mathrm{i}}}^ + }{v}^{0.5},
\end{eqnarray*}

where *I*_p_ is the value of the peak current (A), *n* is the number of transferred electrons (*n* = 2 for LSB), *A* is the area of active material (cm^−2^), *D*_Li*^+^*_ is the diffusion coefficient of Li^+^, *C*_Li^+^_ is the concentration of Li^+^ (mol mL^−1^) and *v* is the scan rate (V s^−1^). The *D*_Li*^+^*_ value was calculated by using a linear fit (Fig. S16 and Table S3). The calculated *D*_Li*^+^*_ of the peak *I*_R2_ of the TB–COF–TFSILi@PE was 9.47 × 10^−8^ cm^2^ s^−1^, which was nearly two times higher than the *D*_Li*^+^*_ values of the TB–COF@PE and TB–COF–SO_3_Li@PE. This enhancement in *D*_Li*^+^*_ suggests that the TB–COF–TFSILi@PE facilitates Li⁺ transport while mitigating polysulfide crossover. Although not a direct indicator of performance, *D*_Li*^+^*_ provides useful insight into the balance between Li⁺ conduction (permeability) and polysulfide shuttle suppression (selectivity), reflecting the significance of the COF@PE design on Li–S battery operation.

### Polysulfide-repellent ability to block the shuttle effect

**Figure 5.**
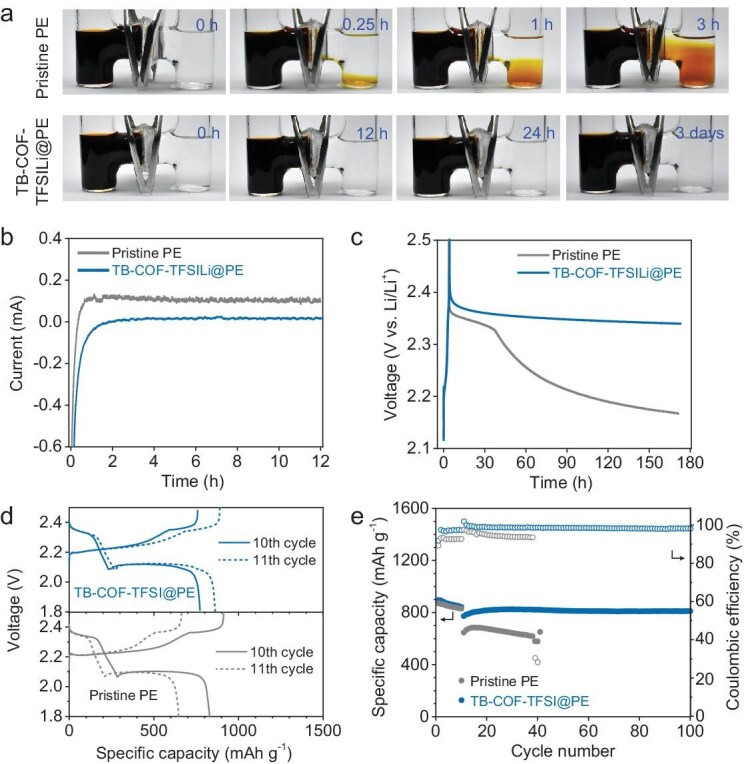
Polysulfide-repellent ability to block the shuttle effect. (a) Digital photographs of polysulfide diffusion of H-type glass cells separated by (top) pristine PE and (bottom) the TB–COF–TFSILi@PE. (b) Shuttle current measurement with the pristine PE and TB–COF–TFSILi@PE without the LiNO_3_ additive. (c) Self-discharge test of charged Li–S cells over a 7-day rest period. The cells were cycled at 0.2 C for 10 cycles and then charged to 2.5 V. (d) Charge/discharge voltage profiles of the cells before and after the rest period (between the 10th and 11th cycles): (top) TB–COF–TFSILi@PE and (bottom) pristine PE. The solid line represents the 10th cycle and the dashed line represents the 11th cycle. (e) Comparison of capacity retention after the self-discharge test with the COF@PEs.

The PS permeability of the TB–COF–TFSILi@PE membranes was investigated to determine its effectiveness in preventing PS crossover (Fig. 5a). For this measurement, Li_2_S_6_ was selected as a representative PS component, considering its contribution to the PSs shuttle effect due to its high solubility in the electrolyte [64]. To this end, 0.05 M Li_2_S_6_ in DOL/DME was prepared in the left chamber, while blank DOL/DME solvent was placed in the right chamber. Within an hour, the pristine PE separator exhibited a vivid jacinth color, indicating the penetration of PSs through the separator at an accelerated rate. After 3 hours, the right chamber was contaminated with vivid scarlet PSs, indicating the failure of the pristine PE to block the penetration of soluble PSs over time. In contrast, there was no distinct change in the color of the TB–COF–TFSILi@PE after 3 days, indicating the effective prevention of PS crossover. Given that conventional separator modification strategies, such as functional coatings incorporating carbon-based or inorganic or hybrid materials, have typically demonstrated PS suppression for ≤24 hours [65,66], this result underscores the excellent polysulfide repulsion capability of the TB–COF–TFSILi@PE, significantly outperforming conventional approaches.

Self-discharge is a major issue of Li–S batteries that is typically caused by the shuttle effect, as it leads to reduced shelf life, inferior electrochemical performance and coulombic efficiency, and depletion of energy [10]. The shuttle currents of COF@PEs were measured to quantify their effectiveness in blocking PS shuttling (Fig. 5b). In this experiment, the current decay correlates with the PS consumption at the Li-metal anode. The cell with the TB–COF–TFSILi@PE exhibited a lower current magnitude of 17 μA after 12 hours compared with that of the pristine PE (100 μA at 2.38 V vs. Li/Li^+^). These results further confirmed the effectiveness of the TB–COF–TFSILi@PE in preventing PS shuttling, as denoted by the negligible conversion of higher-order PS into lower-order PS at the Li-metal anode. The self-discharging phenomena were further investigated by measuring the open circuit voltage (OCV) over time (Fig. 5c). The Li–S cells were galvanostatically charged and discharged at a rate of 0.2 C for 10 cycles, after which they were charged to 2.5 V vs. Li/Li^+^ and left to rest for a week. The OCV of the cell with pristine PE decreased to 2.17 V vs. Li/Li^+^ after a week. In contrast, the cell with the TB–COF–TFSILi@PE exhibited excellent anti-self-discharge performance, as it maintained a high potential of 2.34 V vs. Li/Li^+^. To verify this advantageous effect, the discharge capacities of the cells were compared after 1 week (between the 10th and 11th cycles) to determine the capacity loss possibly caused by the self-discharge (Fig. 5d). The capacity loss of the cell with the pristine PE (22.9%) was significantly higher than that of the cell with the TB–COF–TFSILi (8.3%). During the subsequent cycles, the cell with the TB–COF–TFSILi@PE maintained stable cycle performance (Fig. 5e), whereas the cell with the pristine PE showed rapid capacity decline. The slight increase in the capacity during the subsequent cycles could be attributed to the reactivation of the PSs trapped by the TB–COF–TFSILi@PE, which was in line with the previously reported results [67]. These results confirm the electrochemical viability of the TFSI^−^-containing COF in mitigating the self-discharge problem of Li–S cells.

### Electrochemical performance of the Li–S cells with the TB–COF–TFSILi@PE

**Figure 6.**
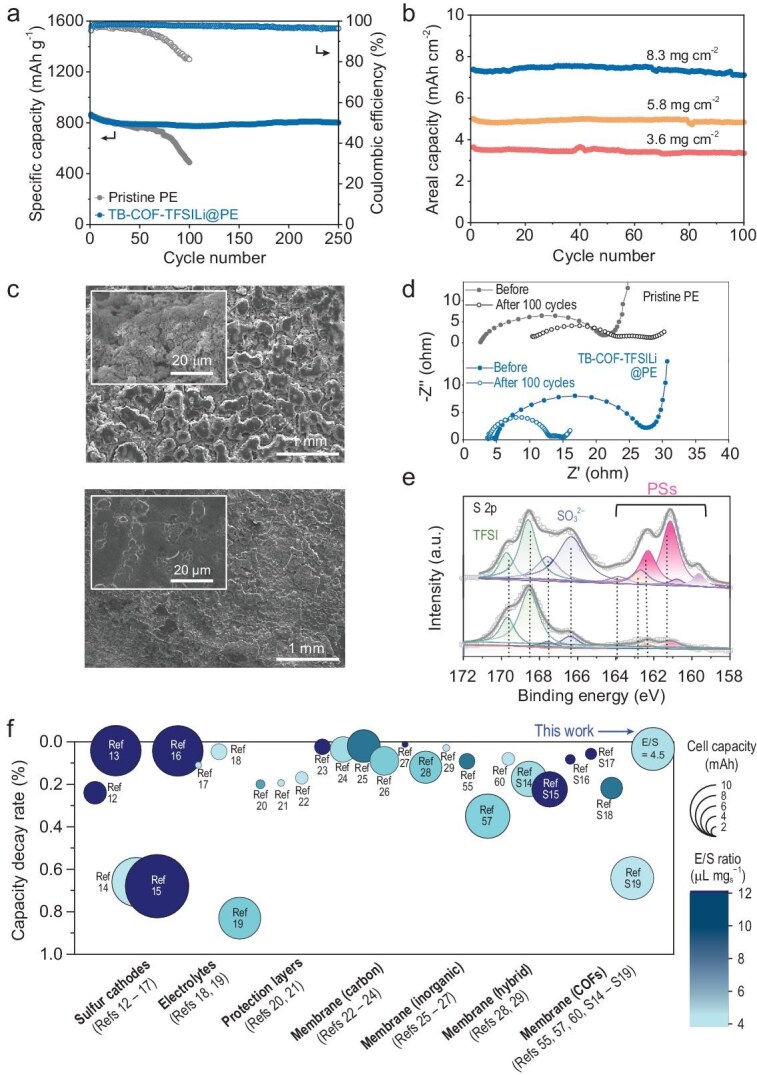
Electrochemical performance of the Li–S cells with the TB–COF–TFSILi@PE. (a) Cycling retention of the Li‒S cells with the TB–COF–TFSILi@PE (vs. pristine PE) at charge/discharge current densities of 0.2 C/0.2 C (S loading = 2.25 mg cm^‒2^; E/S ratio = 10 μL mg_sulfur_^‒1^). (b) Cycling retention of the Li‒S with the TB–COF–TFSILi@PE at charge/discharge current density of 0.1 C/0.1 C and E/S ratio = 4.5 μL mg_sulfur_^‒1^ as a function of S loading. (c) Surface SEM images of the Li-metal anodes after 100 cycles, in which the Li–S cells were fully discharged: (top) pristine PE and (bottom) TB–COF–TFSILi@PE. (d) EIS spectra of the Li‒S cells with (top) pristine PE and (bottom) TB–COF–TFSILi@PE before and after 100 cycles. (e) S 2p XPS spectra and the associated composition analysis of the Li-metal anodes after 100 cycles: (top) pristine PE and (bottom) TB–COF–TFSILi@PE. (f) Comparison of Li–S cell with the TB–COF–TFSILi@PE (this study) and previously reported Li–S cells, with a focus on the capacity decay rate, cell capacity (area) and E/S ratio (heat map).

The cycling performance of the Li–S cells with the pristine PE and TB–COF–TFSILi@PE was evaluated at 0.2 C. After 250 cycles, the cell with the TB–COF–TFSILi@PE exhibited a higher capacity retention of 92.6%, whereas the cell with pristine PE retained only 57.2% of its original capacity after 100 cycles (Fig. 6a). The TB–COF–TFSILi@PE exhibited excellent electrochemical performance, with 0.034% decay per cycle. It should be noted that the decay per cycle of the TB–COF–TFSILi@PE at a discharge rate of 0.2 C is lower than those of previously reported Li–S cells (Table S4). This finding is significant because the capacity degradation rate of Li–S cells typically increases at lower current densities, indicating that the TB–COF–TFSI can effectively mitigate the shuttle effect while facilitating Li⁺. Additionally, the TB–COF–TFSILi@PE achieved superior cycling retention even at a faster current charge/discharge rate of 2.0 C (Fig. S17). To explore the practical feasibility of the TB–COF–TFSILi, Li–S cells were fabricated by pairing thin (50 μm) Li-metal anodes and high-S-loading (ranging from 3.6 to 8.3 mg cm^−2^) cathodes in a lean electrolyte (4.5 μL g_sulfur_^−1^). Under these stringent cell conditions, the cells showed an increase in the areal capacity that was proportional to the S loading, while maintaining stable cycling performance over all of the S loadings (Fig. 6b). Additionally, the applicability of the TB–COF–TFSILi was demonstrated in pouch-type Li–S cells (areal capacity = 4.5 mAh cm^–2^, electrode size = 3 × 3 cm^2^) fabricated under practical yet challenging conditions, including high sulfur loading (5.2 mg cm^–2^), a thin Li-metal anode (50 μm) and a lean electrolyte (4.5 μL g _lf_ᵤᵣ^–1^) (Fig. S18 and Table S6). The resulting pouch cell with the TB–COF–TFSILi@PE exhibited a high cathode capacity of 40.5 mAh along with stable cycling retention. These results underscore the electrochemical reliability of the TB–COF–TFSILi-based membranes, particularly in enhancing the long-term cycling stability of Li–S cells.

The beneficial effect of the TB–COF–TFSILi@PE on the cycling performance was verified by performing a post-mortem analysis of the cycled Li–S cells. The cycled Li-metal anodes (after 100 cycles) were analysed by using SEM images to evaluate the effectiveness of the TB–COF–TFSILi in Li-stripping/plating reversibility. When the pristine PE was used, the cycled Li-metal anode exhibited a porous morphology, with its surface randomly covered with dead Li (Fig. 6c, top). In contrast, the cycled Li-metal anode showed a comparatively smoother morphology when the TB–COF–TFSILi@PE was used (Fig. 6c, bottom). Given that a high Li^+^ transference number promotes uniform Li^+^ diffusion and stable Li deposition by mitigating space-charge formation, the charge/discharge reversibility of Li might be enhanced in the cell containing the TB–COF–TFSILi@PE [68]. To provide additional evidence, the electrochemical impedance spectroscopy (EIS) spectra of the cycled Li–S cells were analysed (Fig. 6d and Fig. S19). After 100 cycles, the bulk electrolyte resistance (*R*_b_) of the TB–COF–TFSILi@PE was negligibly changed, whereas a significant increase in the *R*_b_ value was observed in the pristine PE. The increase in the *R*_b_ of Li–S cells might be due to electrolyte depletion resulting from the formation of Li dendrites and dead Li in the Li anode [69]. Moreover, the electrode−electrolyte interfacial resistance (*R*_int_) and charge-transfer resistance (*R*_ct_) of the TB–COF–TFSILi@PE were lower than those of the pristine PE, indicating the stabilized electrode−electrolyte interface enabled by the TB–COF–TFSILi@PE. Meanwhile, the decrease in the *R*_int_ and *R*_ct_ values after the cycling test could be attributed to the relocation of sulfur active materials during the phase transition [70].

Next, XPS was employed to further elucidate the chemical composition of the cycled Li surface (Fig. 6e). The characteristic S 2p_3/2_ peaks observed at <165 eV can be attributed to the decomposition byproduct of PS, whereas the peaks at >165 eV were derived from TFSI^−^ anions from salts containing oxide or sulfoxide [44,45]. The cell with pristine PE exhibited a significantly stronger peak intensity of the insoluble Li_2_S_2_/Li_2_S layer compared with the cell with the TB–COF–TFSILi@PE. To assess the degree of the parasitic reactions of PSs, the atomic content on Li-metal anodes was investigated by using XPS analysis (Fig. S20) and the results revealed that the atomic S content on the Li-metal anodes with the TB–COF–TFSILi@PE (0.8 at% S) was lower than that on the anode with pristine PE (2.1 at% S). Additionally, the atomic Li content was higher on the Li anode with the TB–COF–TFSILi@PE (43.8 at% Li) than that with pristine PE (29.7 at% Li), indicating the effective inhibition of the parasitic reactions of PSs by the TB–COF–TFSILi@PE. Meanwhile, the structural stability of the TB–COF–TFSILi@PE was analysed before and after 100 cycles (Fig. S21). No significant changes in the FT–IR spectra were observed in the characteristic peaks of νCH₂ and νC = N, demonstrating that the TB–COF–TFSILi maintained its structural integrity during the cycling test.

A comparative analysis with previously reported Li‒S cells was performed to demonstrate the advantageous effects of the TB–COF–TFSILi@PE (Table S5), with a focus on selectivity (capacity decay rate) and permeability (cell capacities and E/S ratios). Maintaining high capacities at low E/S ratios is essential for achieving practical energy-dense Li‒S cells. The TB–COF–TFSILi@PE achieved an exceptionally low rate of capacity decay (0.046%) under stringent cell conditions (8.3 mAh and E/S ratio of 4.5 μL mg_sulfur_^–1^) (Fig. 6b), whereas most of the previous works—including S cathode modifications [12–17], electrolyte formulations [18,19], protective layers [20,21], various membranes [22–29], even those incorporating COFs [55,56,59]—showed high rates of capacity decay under non-practical cell operating conditions (low capacities and high E/S ratios) (Fig. 6f). Given that the reversible electrochemical conversion reaction of S is hindered under lean electrolyte conditions, this study underscores the pivotal role of the TB–COF–TFSILi@PE in facilitating the electrostatic permselective effect, thereby enhancing conversion kinetics and preventing the PS shuttle effect. These advancements collectively contribute to improved electrochemical cycling performance and energy densities in Li‒S cells.

## CONCLUSION

In summary, we have presented the ion channel-gated COF as an ionic diode membrane designed to resolve the conflicting challenge between shuttle prevention and Li^+^ conduction in Li–S batteries. The bulky anion (TFSI^−^)-tethered COF channels delocalized the negative charge sites and reduced the pore size, thereby repelling the PSs through a combination of electrostatic interaction and steric hindrance. Consequently, the TB–COF–TFSILi@PE effectively mitigated the shuttle effect without impairing Li^+^ transport, in contrast to the TB–COF–SO_3_Li@PE. Moreover, the TB–COF–TFSILi@PE alleviated the self-discharge problem and inhibited the parasitic reactions. Owing to these beneficial effects of the bulky anion-tethered COF, the Li–S cells with the TB–COF–TFSILi@PE exhibited improved cycling retention (decay rate = 0.034% per cycle vs. pristine PE separator = 0.428%). Particularly, the TB–COF–TFSILi@PE enabled the realization of practical high-energy-density Li–S cells under constrained conditions. Beyond Li–S batteries, the ion channel-gated COF, with its charge-delocalized and size-tuned pores, holds promise as a platform technology for next-generation rechargeable batteries. Its selective ion-transport mechanism not only suppresses side reactions, but also ensures efficient ion conduction, making it applicable to Li-ion and metal–sulfur batteries, including those based on sodium, potassium, magnesium and aluminum. These properties highlight its potential as a versatile membrane for advanced energy-storage systems.

## Supplementary Material

nwaf193_Supplemental_File

## FUNIDNG

This work was supported by the Basic Science Research Program (RS-2024-00344021, RS-2023-00261543 and 2021M3H4A1A02099355) through the National Research Foundation of Korea (NRF) grant by the Korean Government (MSIT), the National Research Council of Science & Technology (NST) grant by the Korea Government (MSIT) (GTL24011-000), and Korea Institute for Advancement of Technology (KIAT) grant funded by the Korea Government (MOTIE) (RS-2024-00420590, HRD Program for Industrial Innovation). This work was also supported by Yonsei University Research Fund of 2020-22-0536. The computational work was supported by the Supercomputing Center/Korea Institute of Science and Technology Information with supercomputing resources, including technical support (KSC-2022-CHA-0007 to D.-H. S.).
