# Supplementary material for: Ion channel-gated covalent organic framework membrane for sustainable lithium–sulfur batteries
Source: Natl Sci Rev. 2025 May 16;12(7):nwaf193. doi: 10.1093/nsr/nwaf193 (PMC12202149; doi:10.1093/nsr/nwaf193)
Supplement: nwaf193_Supplemental_File [file nwaf193_supplemental_file.pdf]

RESEARCH ARTICLE

MATERIALS SCIENCE

Ion channel-gated covalent organic framework membrane for sustainable lithium-sulfur batteries

Zhongping Li[1,2,†], Jae-Seung Kim[3,4,†], Hyunseok Moon[2,†], Kyeong-Seok Oh[2], Yuxin Hou[5], Sodam Park[4,*], Kun Ryu[5], Changqing Li[4], Jeong-Min Seo[4], Xiaoming Liu[5], Jong-Beom Baek[4], Dong-Hwa Seo[6,*] and Sang-Young Lee[2,7,*]

[1]Key Laboratory of Automobile Materials of MOE and School of Materials Science and Engineering, Jilin University, Changchun 130012, China;

[2]Department of Chemical and Biomolecular Engineering, Yonsei University, Seoul 03722, South Korea;

[3]Department of Materials Science and Engineering, Korea Advanced Institute of Science and Technology (KAIST), Daejeon 34141, South Korea;

[4]School of Energy and Chemical Engineering, Ulsan National Institute of Science and Technology (UNIST), Ulsan 44919, South Korea;

[5]College of Chemistry, Jilin University, Changchun 130012, China;

[6]Pritzker School of Molecular Engineering, The University of Chicago, Chicago, IL, 60637, USA;

[7]Department of Battery Engineering, Yonsei University, Seoul 03722, South Korea

***Corresponding authors**. E-mails: souldotcom@unist.ac.kr; dseo@kaist.ac.kr; syleek@yonsei.ac.kr

[†]Equally contributed to this work.

METHODS

Materials

Dioxane, mesitylene, acetic acid, lithium chloride, tetrahydrofuran, copper(I) iodide, and othe rs chemicals were purchased from Sigma Aldrich, Tokyo Chemical Industry Co., Ltd, FUJIFI LM Wako, and other companies. 2,5-Bis (prop-2-yn-1-yloxy) terephthalaldehyde (BPTA), 2,4,6-tris(4-aminophenyl)-1,3,5-triazine (TAPT), lithium 3-azidoproanesulfonyltrifluoromethanesulfonylimide (N_3-TFSILi), and 3-azidopropanesulfonic acid sodium salt (N_3-SO_3Na) were synthesized as previously reported[1-4].

Characterization

Fourier transform infrared spectra were collected using a Bruker ALPHA Laser class 1. Powder X-ray diffraction (PXRD) patterns were recorded on a Rigaku D/MAX2500 operated at 40 kV and 200 mA with Cu Kα radiation (λ = 1.54056 Å) at a range of 2θ = 2–35 deg. (2 deg. min^{-1}). Thermogravimetric analysis (TGA) was performed on a TA Instruments Q500 thermogravimetric analyzer under a nitrogen atmosphere with a heating rate of 10 ℃ min^{-1}. Solid-state nuclear magnetic resonance (NMR) experiments were conducted using an Agilent VNMRS 600 MHz NMR spectrometer at room temperature. Nitrogen sorption isotherms were measured at 77 K with a TriStar II instrument (Micromeritics). The Brunauer-Emmett-Teller (BET) method was utilized to calculate the specific surface areas. By using the non-local density functional theory (NLDFT) model, the pore size was derived from the sorption curve. Morphology images were characterized with a Zeiss Merlin Compact field emission scanning electron microscope (SEM) at an electric voltage of 15 KV. As a model compound, the samples were soaked with electrolyte (1.0 M LiTFSI in DOL/DME) before conducting the ^7Li NMR test. The chemical shift of Li salts was recorded by using an NMR (600 MHz FT-NMR, VNMRS 600 (Agilent)) with 1.6 mm HXY Fast MAS T3 probe. The chemical shift is referenced to a 1.0 M aqueous LiCl solution at ^7Li (0.0 ppm) as an external standard. The binding energy was analyzed X-ray photoelectron spectroscopy (XPS, ThermoFisher) with focused monochromatized Al Kα radiation. Cross-sectioned membrane sample was investigated by using Cross Section Polisher (IB-19510CP, JEOL). Cyclic valtammetry (CV) was also analyzed by VSP calssic, Bio-Logic). All the cell performance was investigated using a cycle tester (PNE Solution) at various charge/discharge conditions. 0.05 M Li_2S_6 in DOL/DME (v/v = 1/1) solution was prepared by dissolving sulfur (S) and lithium sulfide

(Li_2S) with the molar ratio of 1:5. Contact angle measurements were performed using a Dataphysics OCA15EC system. In this method, a 5 μL droplet of electrolyte was dispensed onto the sample surface using a syringe, and the contact angle was subsequently measured.

Electrochemical measurement

Ionic conductivities were measured using Li^+ blocking titanium SUS/COF@PE/SUS symmetric cell employing electrochemical impedance spectroscopy (EIS) analysis in a frequency range of 10^{-2} to 10^6 Hz with an applied amplitude of 10 mV using a Bio-logic VSP classic potentiostat. The ionic conductivities (σ) were determined using the following equation:

$$\sigma = \frac{l}{RA}$$

where l represents the pellet thickness, R is the resistance, and A is the area in contact with the electrodes. The Li^+ transference numbers ($t_{Li□}$) were evaluated using a potentiostatic polarization method. By analyzing the DC polarization through a Li^+ non-blocking symmetric cell and its sequential EIS before and after polarization, the Li^+ transference number was calculated using the formula:

$$t_{Li+} = \frac{I_s(\Delta V - I_o R_o)}{I_o(\Delta V - I_s R_s)}$$

,where ΔV is applied potential, I_o and R_o represent the initial current and resistance, and I_s and R_s denote the steady-state current and resistance after the polarization, respectively. The sulfur cathodes, with areal sulfur loading of approximately 1.0, 1.8 and 2.25 mg cm^{-2}, were prepared using a composition ratio of S-CNT composite/MWCNT/xanthan gum binder = 80/10/10 (w/w/w), followed by vacuum drying for one day. The S-CNT composite was prepared by mixing sublimated sulfur and MWCNT in a mass ratio of 7:3 and heating the mixture at 155 °C for 12 h in a sealed tube. High mass loading sulfur cathodes (3.6, 5.8, and 8.3 mg cm^{-2}) were prepared using the same method. A coin-type (CR2032) cell was fabricated by assembling the sulfur cathode, and lithium foil anode in an argon-filled glove box. 1.0 M LiTFSI in DOL/DME (1/1 = v/v) with 2 wt% $LiNO_3$ additive was used as a liquid electrolyte for the whole cell test. The cyclic voltammetry was conducted using Li–S cell under a sweep rate of 0.1 mV s^{-1} within a voltage range of 1.65 to 2.65 V (vs. Li/Li^+). For the

rate capability test, cells were cycled at discharging current rates ranging from 0.1 to 5.0 C. For shuttle current measurement, cells were assembled without adding $LiNO_3$. During the cycling process at 0.2 C, shuttle current of cells was detected by potentiostatic hold on discharge at 2.38 V. For the post-mortem analysis, cycled cells were disassembled and rinsed with dimethyl ether (DME) to remove residual salts. The whole fabrication process of the cells was carried out in a dry room at room temperature. The various charge/discharge conditions were applied under a voltage range of 1.8−2.5 V.

Density functional theory (DFT) calculations for simulated XRD generation.

Computational calculations for generating COF structures with XRD patterns were performed using the Vienna Ab initio Simulation Package (VASP) with following settings. The generalized gradient approximation (GGA) exchange-correlation with the Perdew-Burke-Ernzerhof (PBE) functionals was utilized to describe potentials, and the projector-augmented wave (PAW) method was employed. To account for van der Waals interactions, dispersion corrections were included using the DFT-D3 method proposed by Grimme. A plane-wave cut-off energy of 520 eV and reciprocal-space k-point meshes of $1 \times 1 \times 2$ were applied in the calculations. The continuous hexagonal repeating structures of the COF were generated by considering one-third of the hexagonal structure of the COF in a single unit cell. For each structure, the cell shape, cell volume, and atomic positions were fully relaxed until the forces on each atom were below 0.05 eV/Å.

Molecular electrostatic potential and interaction energy calculation.

The DFT calculations were performed using the Gaussian09 software package. Becke-Lee–Yang–Parr (B3LYP) hybrid exchange–correlation functional with 6-311G(d,p) basis sets for all atoms are used for the geometrical and energy evaluation. To describe the polarity of the utilized electrolyte (1.0 M LiTFSI in DOL/DME (v/v = 50/50) + 2.0 wt% $LiNO_3$), a polarizable continuum model (PCM) solvation model with a solvent dielectric constant (ε = 7.103) was applied to all processes. Natural Bond Orbitals (NBOs) charge in Gaussian09 was used to generate molecular electrostatic potential (MESP). In order to quantify the difference between a complex state and an individual molecular state, the Gibbs free energy of binding (ΔG_b) was defined as follows:

$$\Delta G_b = E_{complex} - E_{COF} - E_{ion},$$

where $E_{complex}$, E_{COF}, and E_{ion} represent the energies of the COF motif-ion complex, the COF motif, and the ion, respectively. Noncovalent interactions (NCI) analysis is conducted by multiwfn software to plot 3D structure of interactions.

Fabrication of COF@PE

The synthetic procedures for TB-COF, TB-COF-SO_3Li, and TB-COF-TFSILi are described in Supplementary Notes 1-3. For membrane preparation, 30 mg of COF was dispersed in deionized (D.I.) water (30 mL) using bath sonication for 20 min at RT. After dispersion, the solution was centrifuged at 3000 g for 10 min. The COF@PE membranes were fabricated by vacuum filtration of the supernatant from the COF dispersion. To enhance surface wettability, the PE membrane was pretreated with UV-ozone (UVO) exposure before the vacuum filtration process. After filtration, the membranes were vacuum-dried at 60 ℃ for 12 h and subsequently punched into 19 mm diameter discs for use in coin cell assembly.

Supplementary Note 1 | Synthesized of TB-COF

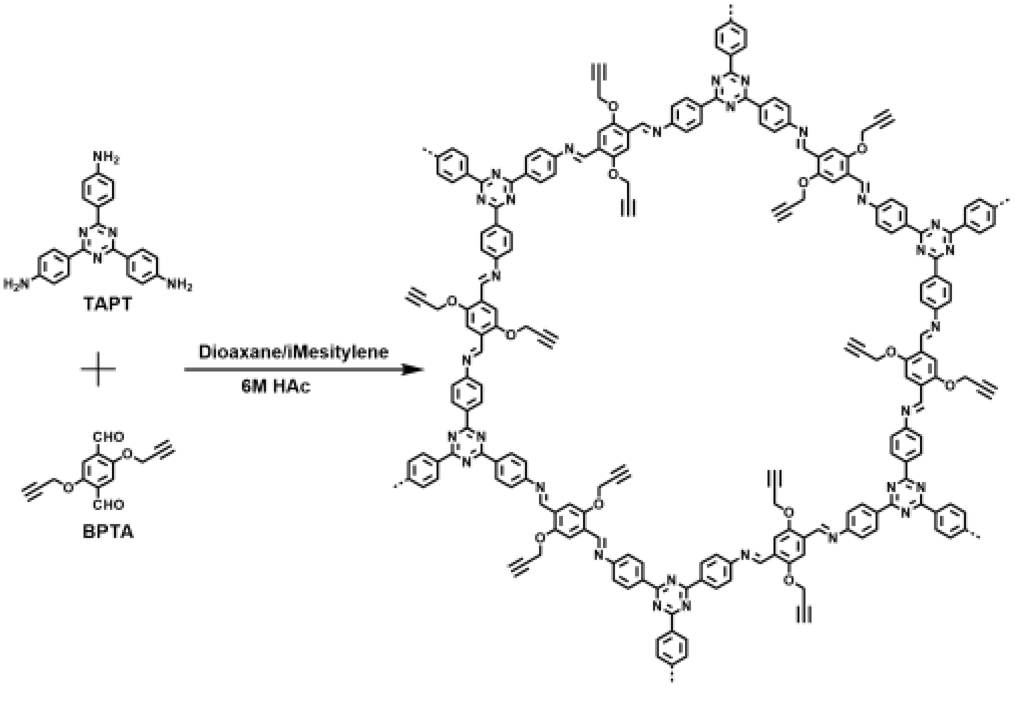

A pyrex tube (10 mL) was charged with BPTA (0.3 mmol), TAPT (0.2 mmol), and 2.4 mL

dioxane/mesitylene (v/v, 1:3), and 0.3 mL of 6 M aqueous acetic acid. The tube was subsequently flash-frozen at 77 K and degassed by three freeze-pump-thaw cycles. The tube was sealed off and then heated at 120 ℃ for 3 days. The powder collected was collected by filtration, washed with N, N'-dimethylacetamide, tetrahydrofuran, and methanol for several times, and soxhleted using tetrahydrofuran overnight. The obtain powder was dried at 100 ℃ under vacuum for 12 hours yielding the yellow TB-COF sample in 85% isolated yield.

Supplementary Note 2 |Synthesized of TB-COF-SO_3Li

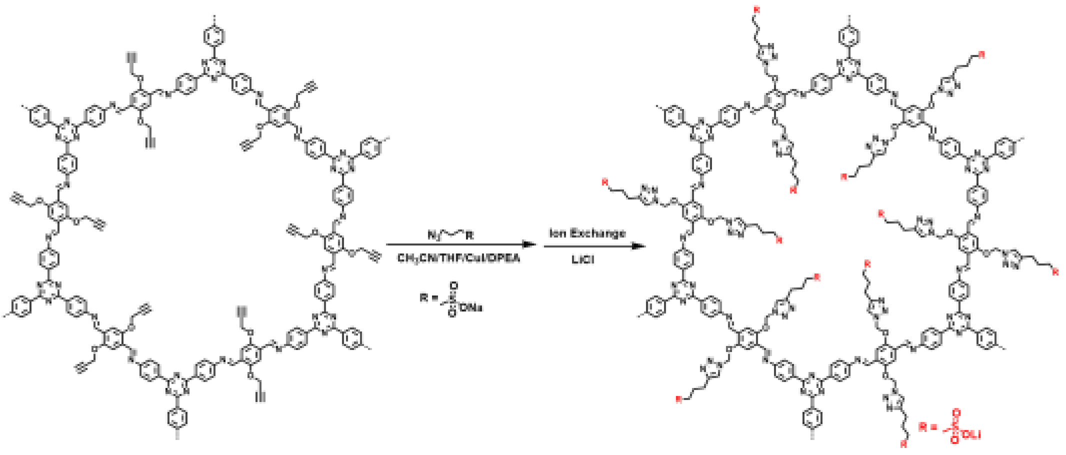

A pyrex tube (10 mL) is charged with BT-COF (100 mg), 3-azidopropanesulfonic acid sodium salt (1.19 mmol), CuI (4.3 mg), N, N-diisopropylethylamine (50 μL), CH_3CN (1 mL), and THF (1 mL). The tube was rapidly frozen at 77 K and degassed by three freeze-pump-thaw cycles. The tube was sealed off and then heated at 50 ℃ for 2 days under nitrogen atmosphere. The powder collected was collected by filtration, and washed with DMF, THF, and methanol for several times. Subsequently, it was dried under vacuum at 50 °C for 12 hours. The deep-organic powder was dispersed and stirred in an aqueous LiCl solution (5 M, 100 mL) at room temperature for ion-exchange. The aqueous LiCl solution was refreshed every 24 hours for a total of five cycles. The precipitate was collected by centrifugation, washed with deionized water, N, N'-dimethylacetamide, and ethanol for five times, and then dried under vacuum at 100 ℃ for 24 hours. This process yielded the BT-COF-SO_3Li product, represented as a deep-organic powder.

Supplementary Note 3 |Synthesized of TB-COF-TFSILi

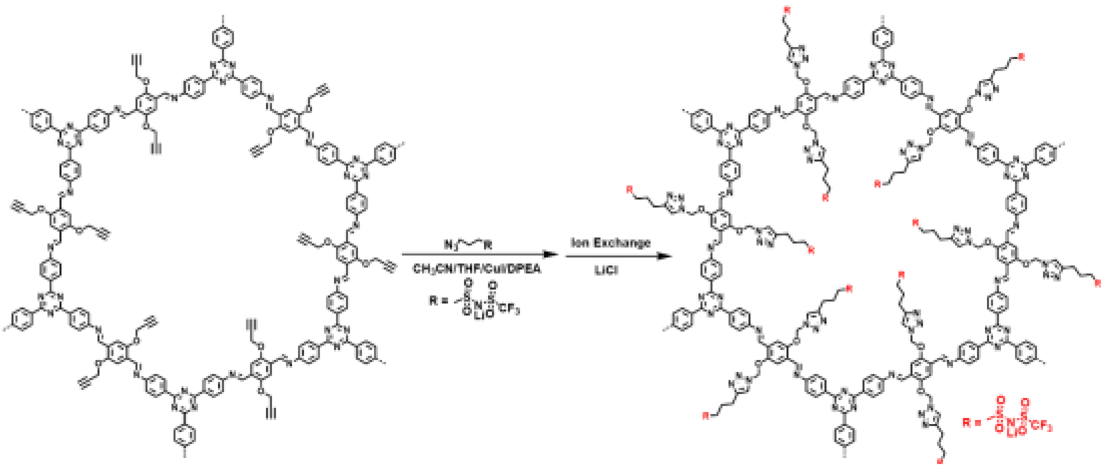

A pyrex tube (10 mL) is charged with BT-COF (100 mg), lithium 3-azidoproanesulfonyltrifluoromethanesulfonylimide (1.19 mmol), N, N-diisopropylethylamine (50 μL), CuI (4.3 mg), CH_3CN (1 mL), and THF (1 mL). The tube was rapidly frozen at 77 K and degassed by three freeze-pump-thaw cycles. The tube was sealed off and then heated at 50 ℃ for 2 days under nitrogen atmosphere. The powder was collected by filtration, and washed with DMF, THF, and methanol for several times, and then dried at 50 ℃ under vacuum for 12 hours. The obtained deep-organic powder was dispersed and stirred in an aqueous LiCl solution (5 M, 100 mL) at room temperature for ion-exchange. To ensure effectiveness, a fresh aqueous LiCl solution was introduced every 24 hours for a total of five cycles. The precipitate was collected by centrifugation, washed with deionized water, DMF, and ethanol for five times, and then dried under vacuum at 100 ℃ for 24 hours to give TB-COF-TFSILi (deep-organic powder).

Supplementary Figures

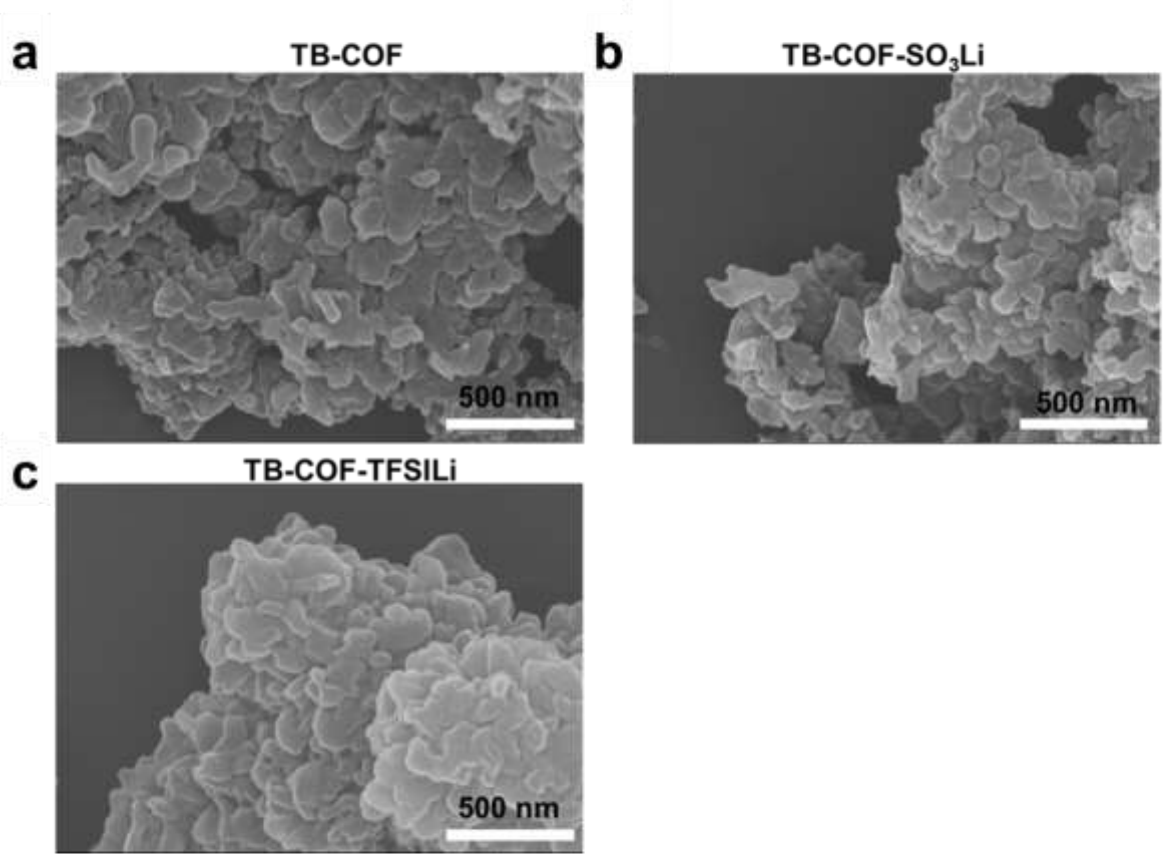

Supplementary Fig. 1 | FE-SEM images of (**a**) TB-COF, (**b**) TB-COF-SO_3Li, and (**c**) TB-COF-TFSILi.

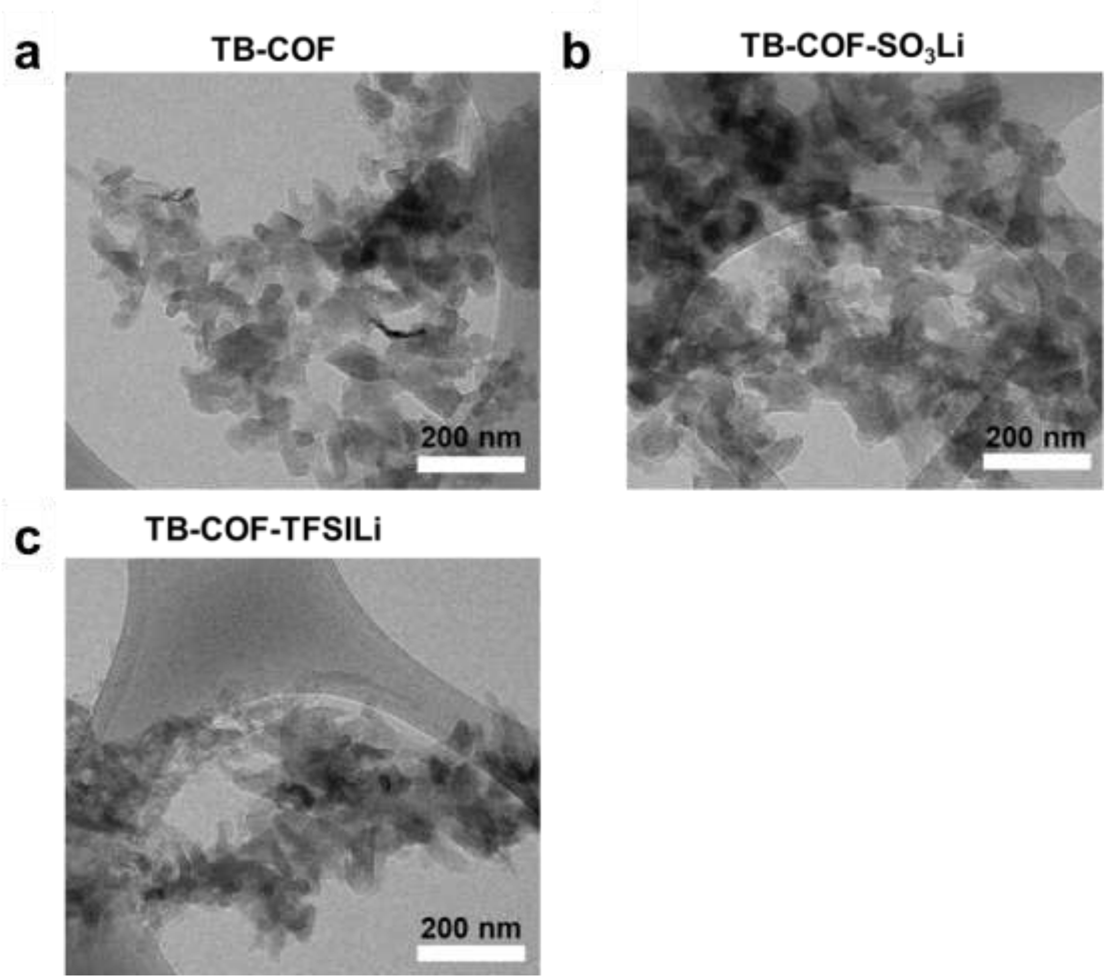

Supplementary Fig. 2 | TEM images of (**a**) TB-COF, (**b**) TB-COF-SO_3Li, and (**c**) TB-COF-TFSILi.

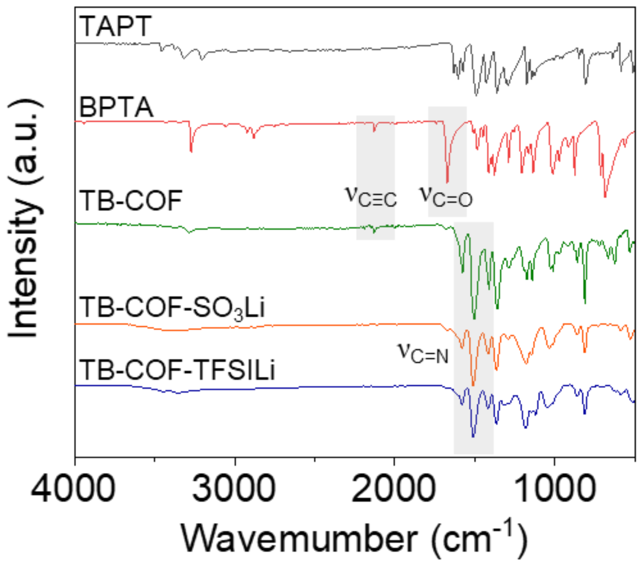

Supplementary Fig. 3 | FT-IR spectra of monomers and COFs.

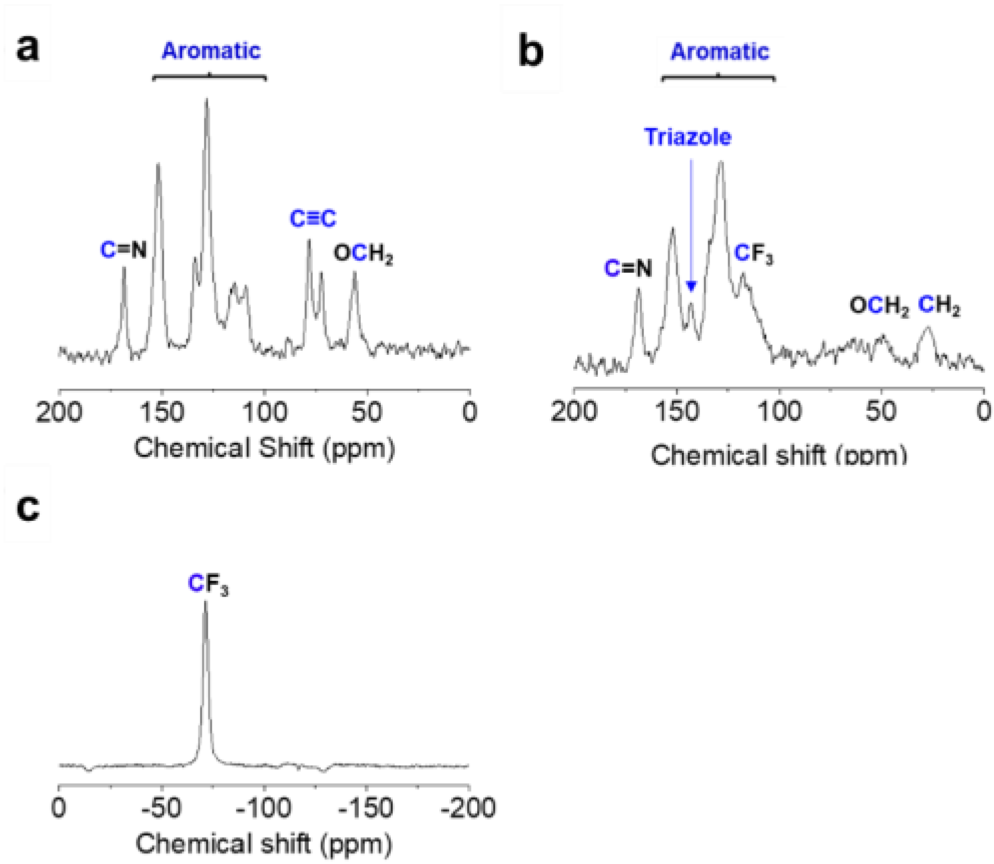

Supplementary Fig. 4 | Solid-state ^{13}C NMR spectra of (**a**) TB-COF and (**b**) TB-COF-TFSILi. (**c**) Solid-state ^{19}F NMR spectrum of TB-COF-TFSILi.

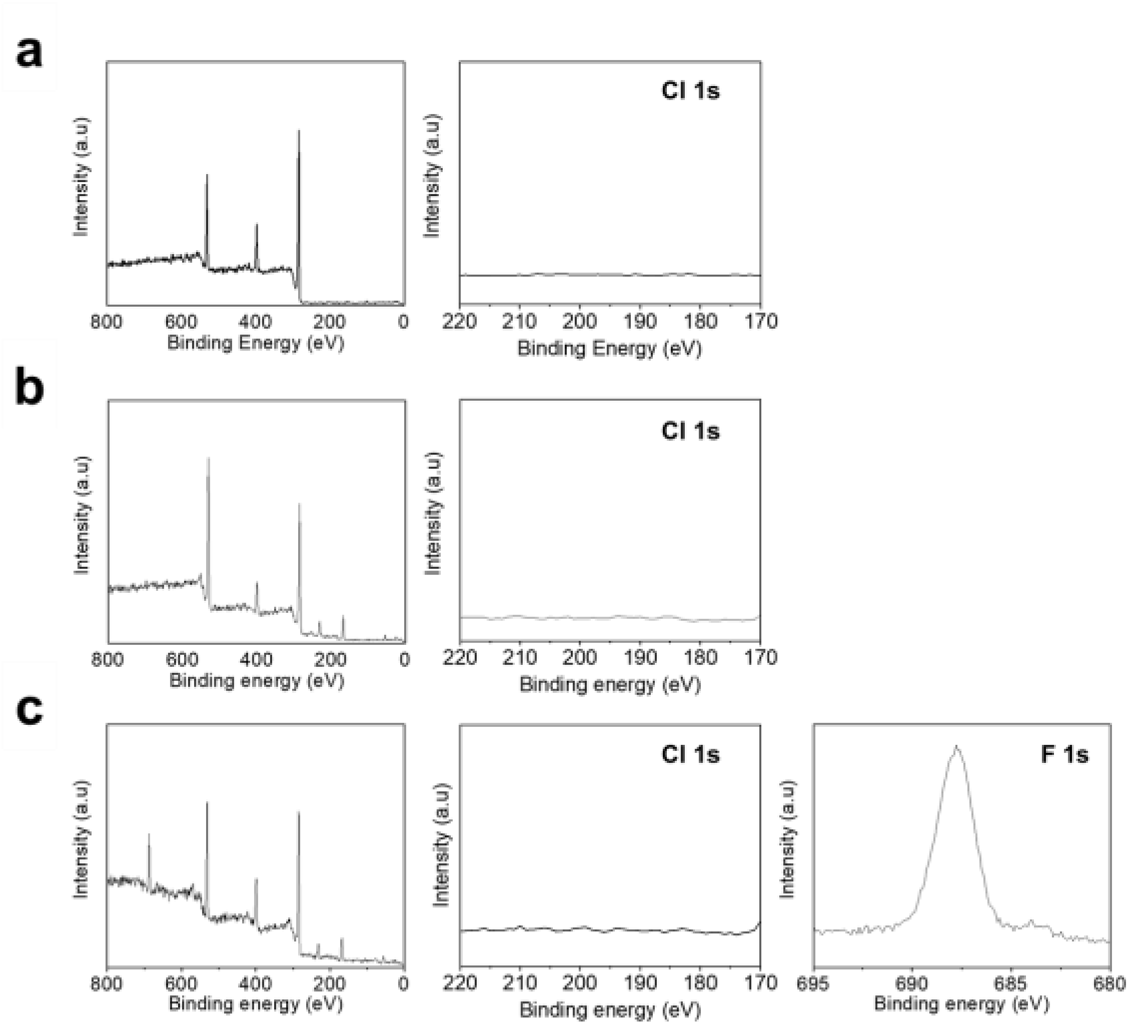

Supplementary Fig. 5 | XPS spectra of (**a**) TB-COF, (**b**) TB-COF-SO_3Li, and (**c**) TB-COF-TFSILi.

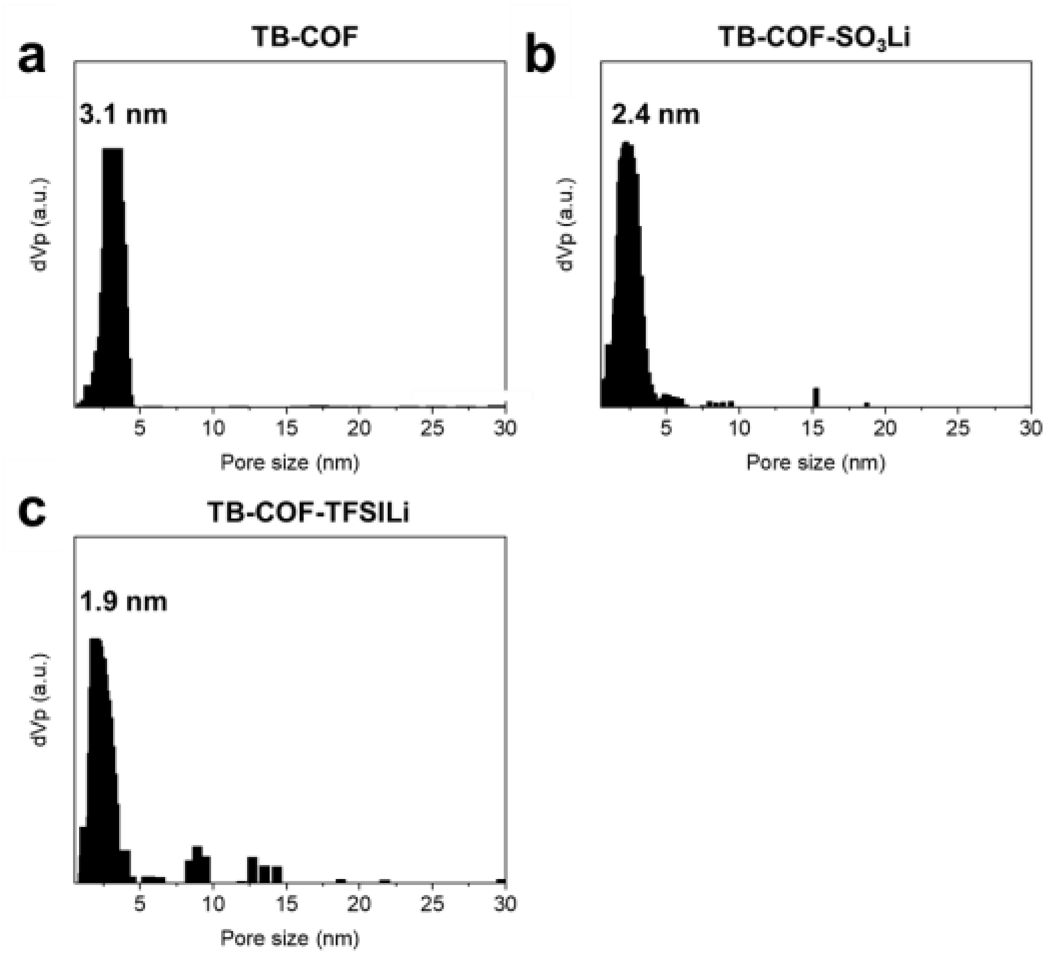

Supplementary Fig. 6 | Pore size distribution (obtained using N_2 adsorption-desorption isotherms) of (**a**) TB-COF, (**b**) TB-COF-SO_3Li, and (**c**) TB-COF-TFSILi.

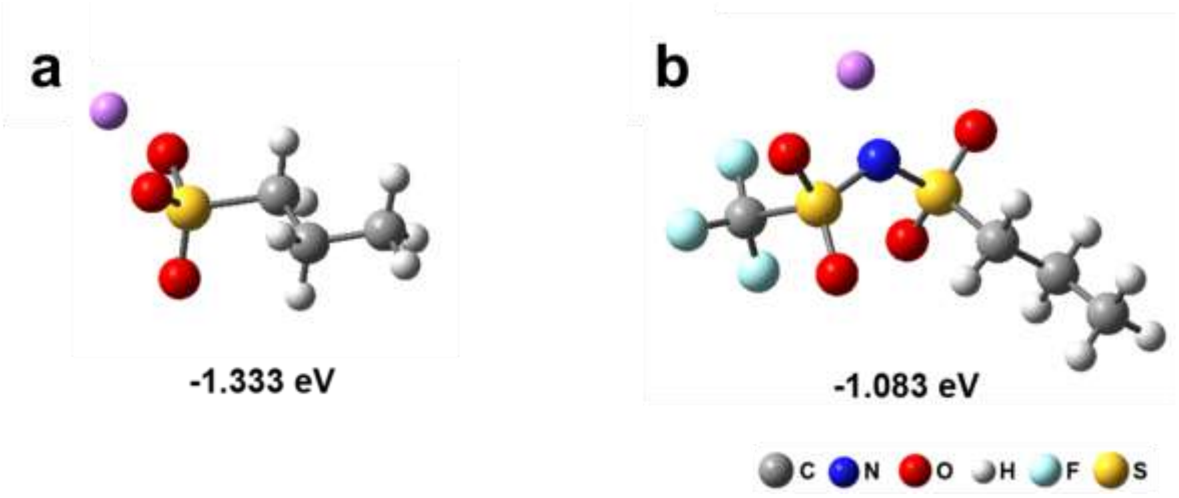

Supplementary Fig. 7 | Structural motif of the functional group for binding energy between Li^{+}: (**a**) TB-COF-SO_3Li and (**b**) TB-COF-TFSILi.

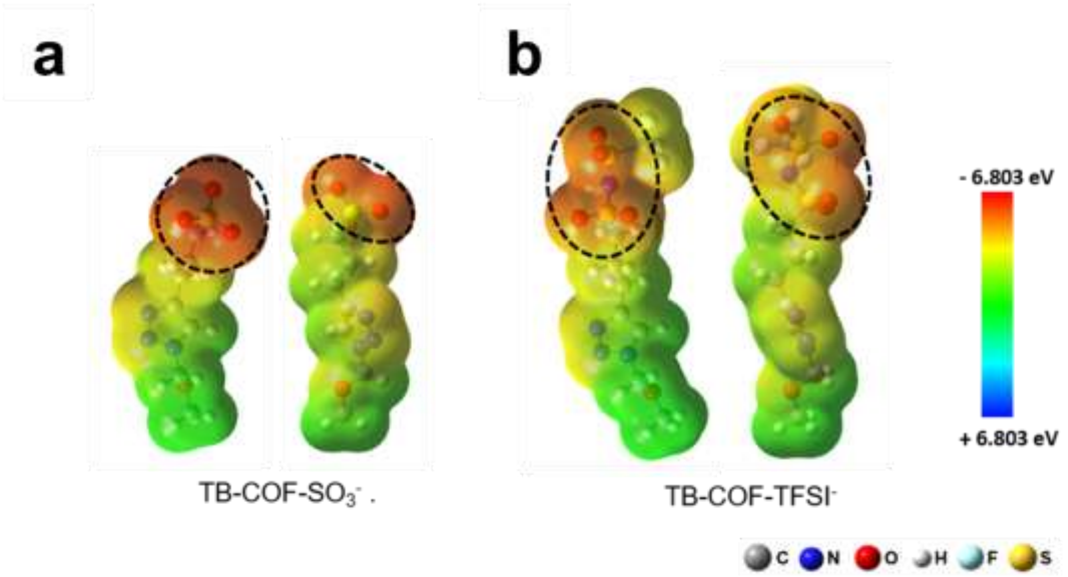

Supplementary Fig. 8 | The molecular electrostatic potential (MESP) plot of the molecular structures of functional group in (**a**) TB-COF-SO_3^{-} and (**b**) TB-COF-$TFSI^{-}$.

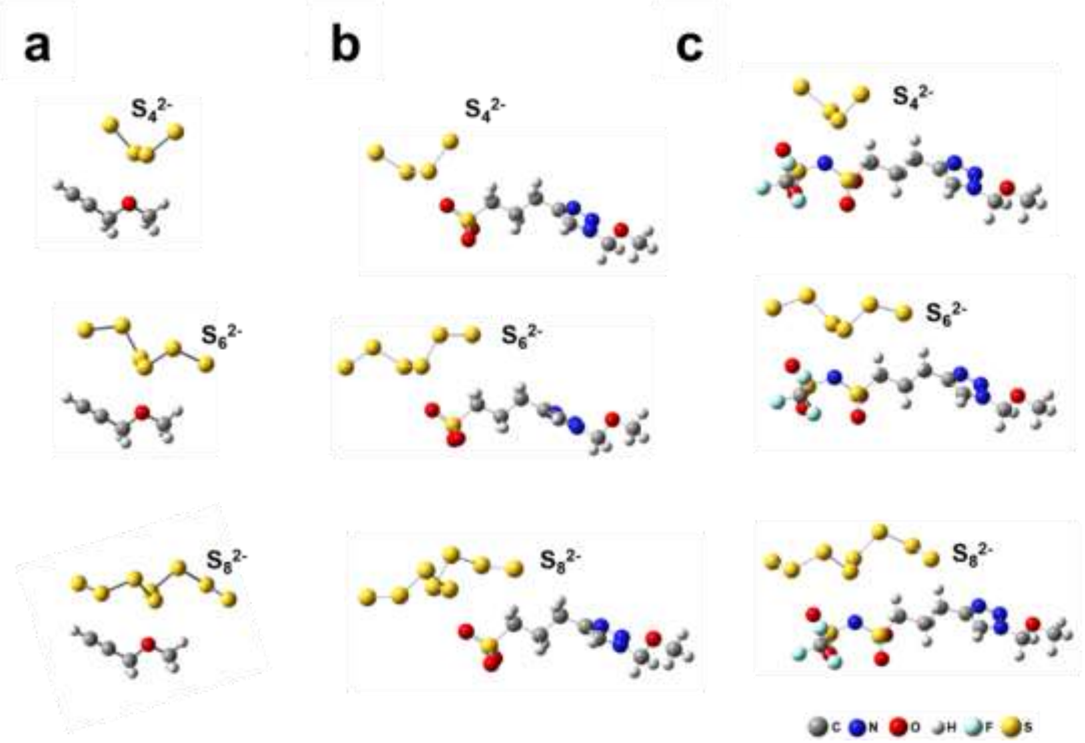

Supplementary Fig. 9 | Motifs for binding energy between different polysulfides (S_4^{2-} (top), S_6^{2-} (middle) and S_8^{2-} (bottom)) and (**a**) TB-COF, (**b**) TB-COF-SO_3^- and (**c**) TB-COF-TFSI$^-$, for comparative analysis.

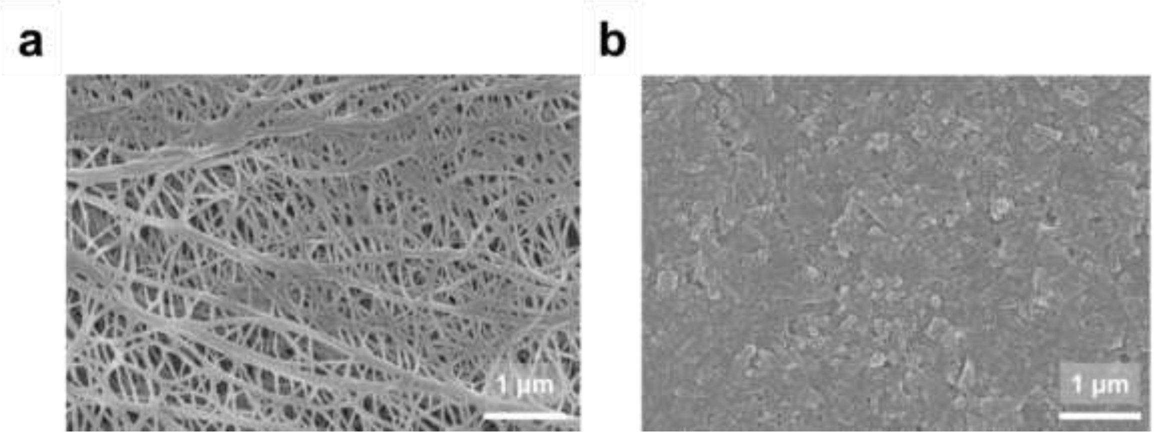

Supplementary Fig. 10 | SEM images of (**a**) pristine PE and (**b**) TB-COF-TFSILi@PE.

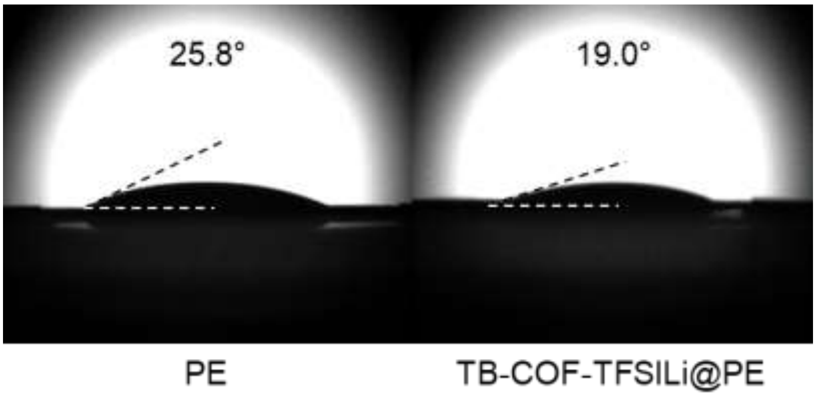

Supplementary Fig. 11 | Electrolyte contact angle of the pristine PE and TB-COF-TFSILi@PE.

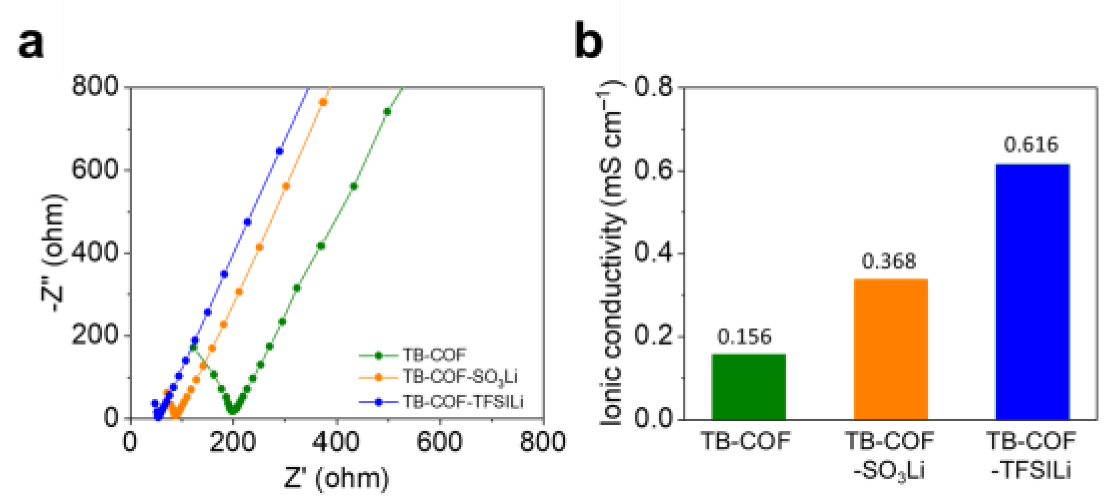

Supplementary Fig. 12 | Ion conduction behavior of the COF pellets (TB-COF, TB-COF-SO_3Li, and TB-COF-TFSILi): (**a**) EIS spectra and (**b**) ionic conductivity, in which the COF pellets were soaked with a liquid electrolyte (1 M LiTFSI in DOL/DME = 1/1 (v/v)) prior to this measurement.

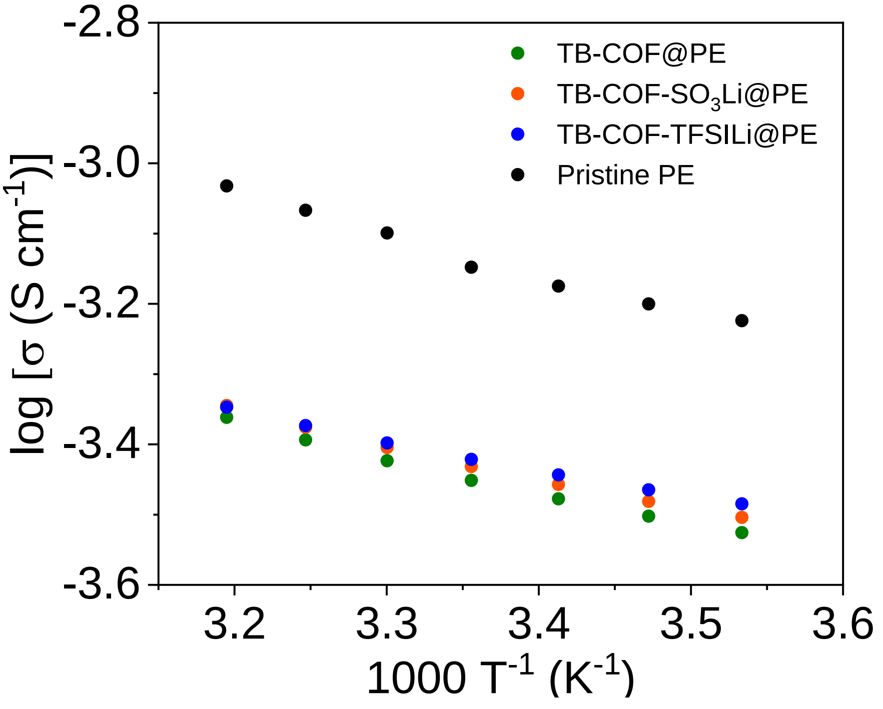

Supplementary Fig. 13 | Ionic conductivities of various COF@PEs.

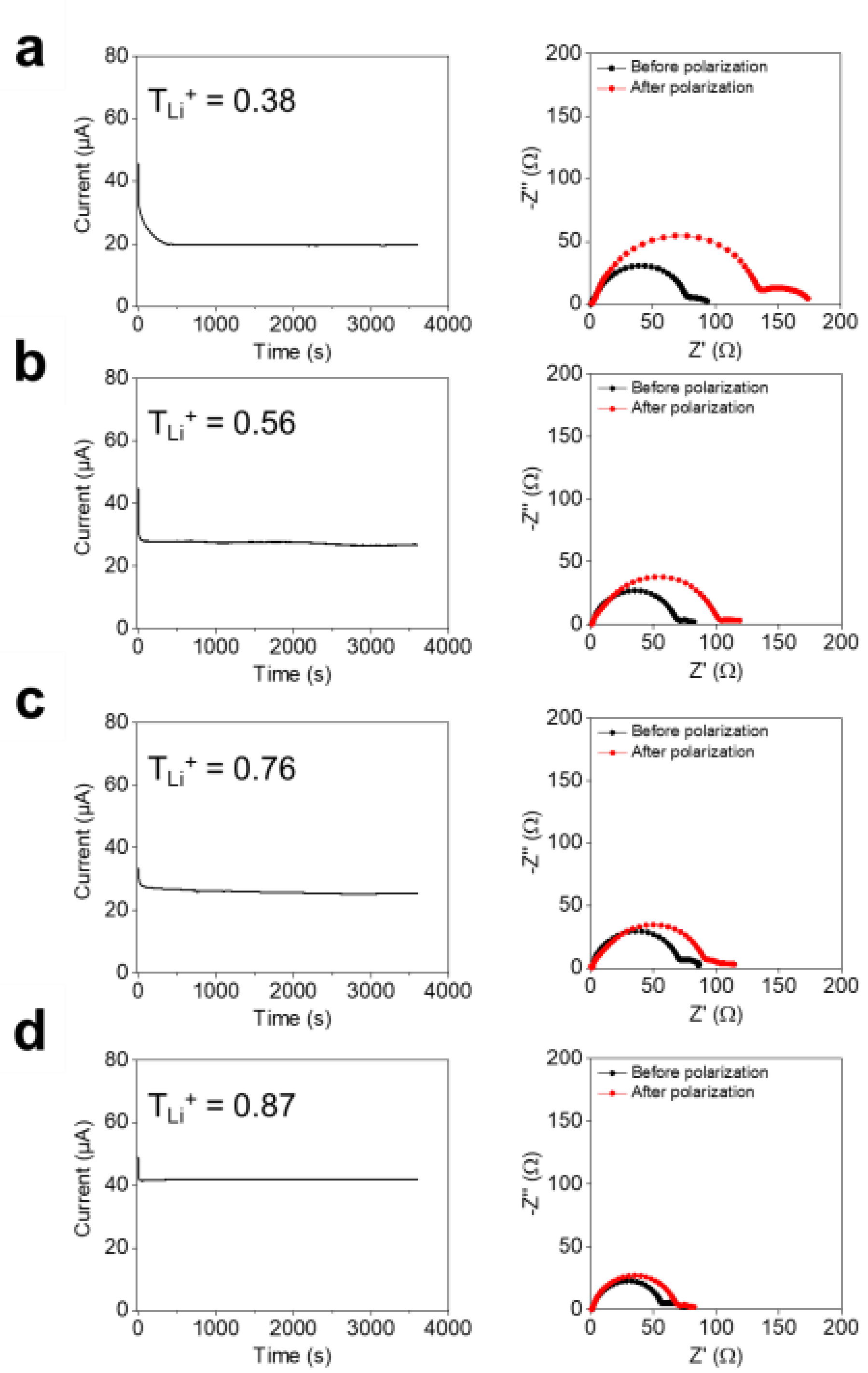

Supplementary Fig. 14 | Time-dependent current profiles (left) and EIS profiles (right) for the Li||Li symmetric cell at 10 mV polarization of liquid electrolyte (1 M LiTFSI in DOL/DME (1/1, v/v) + 2wt% $LiNO_3$) impregnated into (**a**) pristine PE, (**b**) TB-COF, (**c**) TB-COF-SO_3Li, and (**d**) TB-COF-TFSILi.

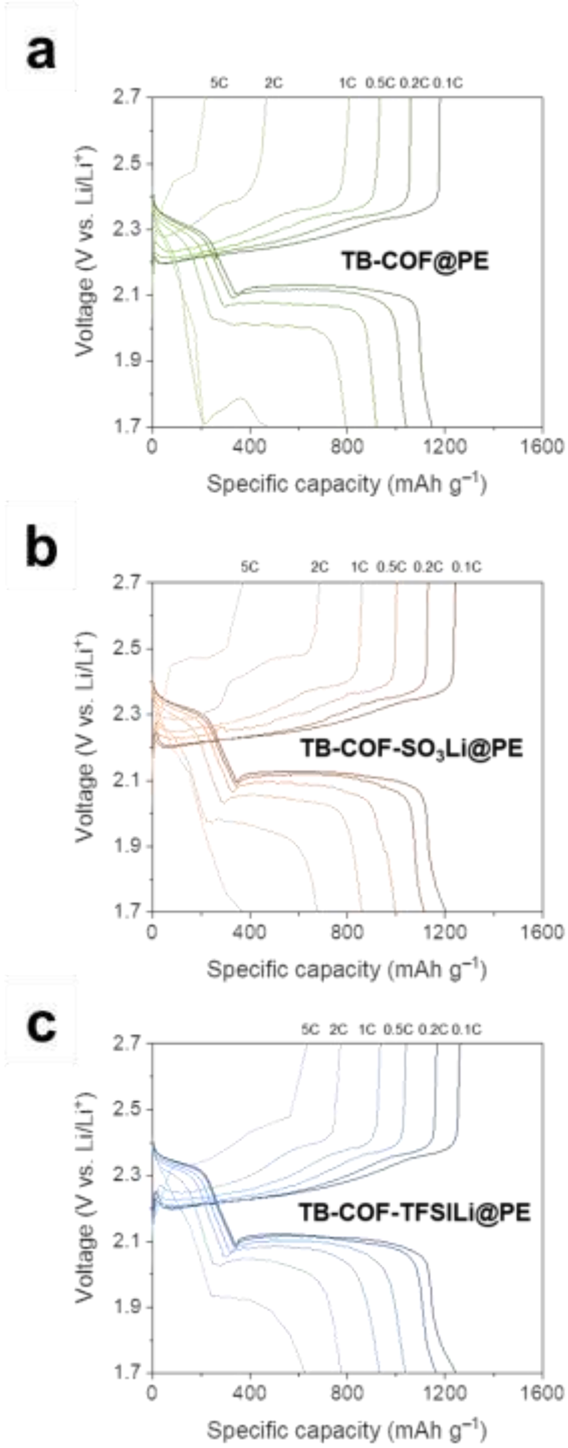

Supplementary Fig. 15 | Voltage profiles of the Li–S cells containing (**a**) the TB-COF@PE, (**b**) TB-COF-SO_3Li@PE, and (**c**) TB-COF-TFSILi@PE (sulfur loading = 1.0 mg cm^{-2}; low E/S ratio = 10 μL mg_{sulfur}^{-1}) at different charge/discharge current rates of 0.1, 0.2, 0.5, 1, 2, and 5 C.

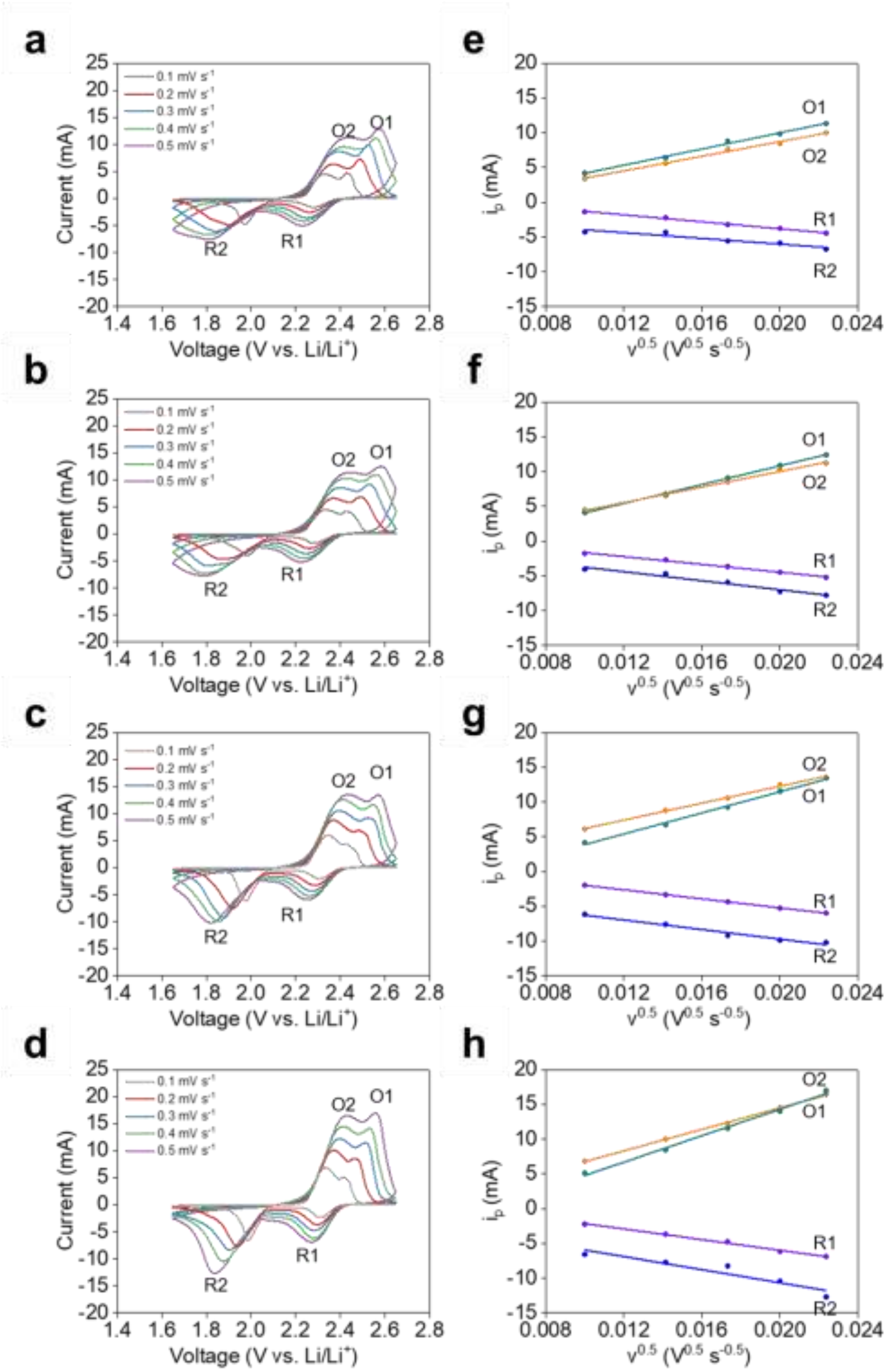

Supplementary Fig. 16 | Cyclic voltammograms at different voltage scan rates of Li–S cells: (**a**) using pristine PE, (**b**) incorporating TB-COF, (**c**) utilizing TB-COF-SO_3Li and (**d**) employing TB-COF-TFSILi. (**e-h**) the linear fits of the peak currents for cells with (**e**) pristine PE, (**f**) TB-COF, (**g**) TB-COF-SO_3Li and (**h**) TB-COF-TFSILi.

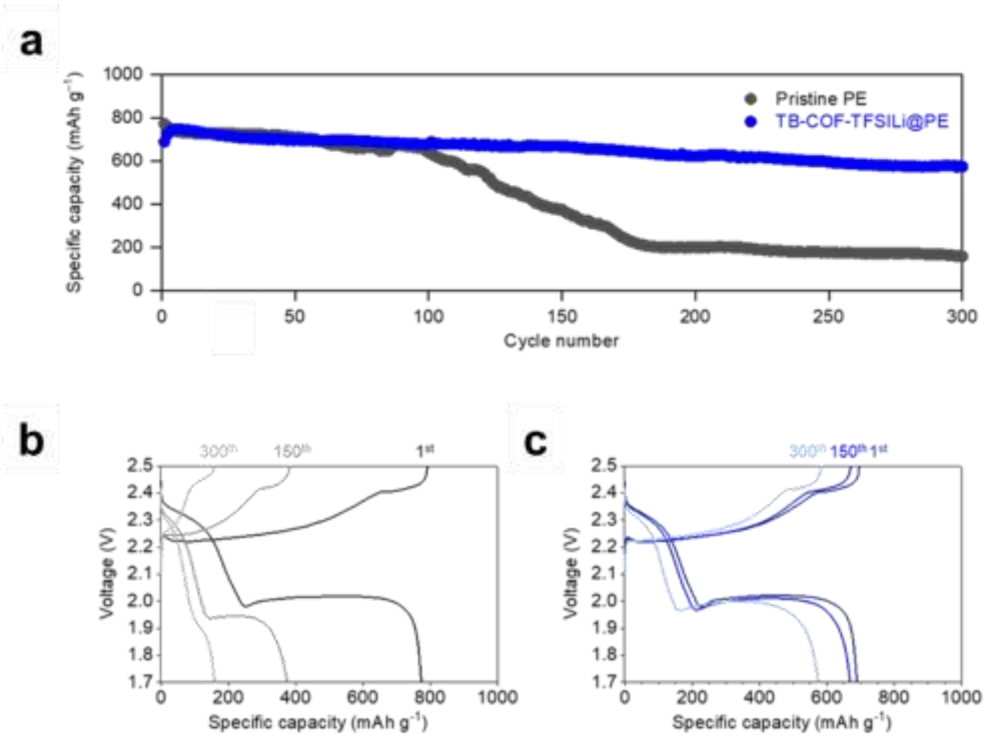

Supplementary Fig. 17 | (**a**) Cycling performance of the Li–S cell with the TB-COF-TFSILi@PE (vs. pristine PE) at a faster charge/discharge C-rate of 2.0 C/2.0 C (sulfur loading = 1.0 mg cm^{-2}; E/S ratio = 10 µL mg_{sulfur}^{-1}). Charge/discharge profiles of (**b**) pristine PE and (**c**) TB-COF-TFSILi@PE.

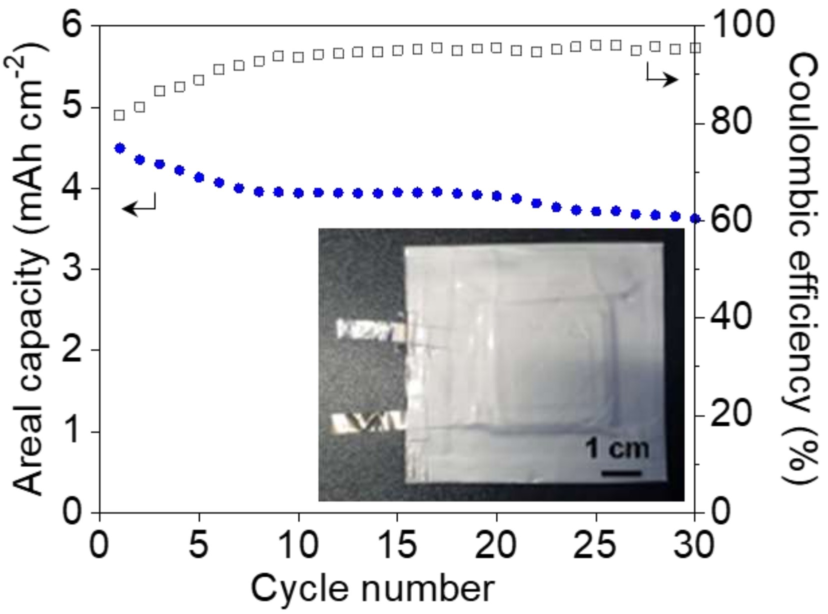

Supplementary Fig. 18 | Cycling retention of the pouch-type Li–S full cell with TB-COF-TFSILi@PE at charge/discharge current densities of 0.05 C/0.05 C under stringent conditions (high sulfur loading (5.2 mg cm^{-2}), thin Li-metal anode (50 μm), and lean electrolyte (4.5 μL g_{sulfur}^{-1})).

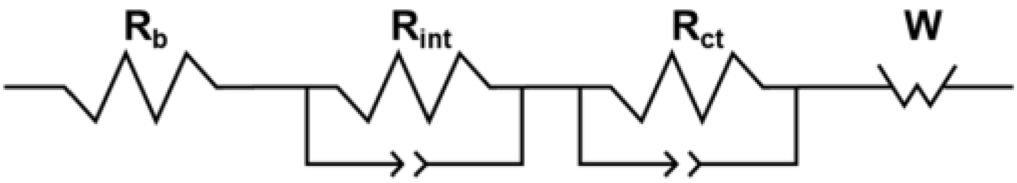

	Before (PE)	After 100 cycles (PE)	Before (TB-COF-TFSI@PE)	After 100 cycles (TB-COF-TFSI@PE)
R_b	2.7	10.4	4.5	3.6
R_{int}		12.9		9.3
R_{ct}	18.7	4.7	21.9	1.9

Supplementary Fig. 19 | Equivalent circuit model and fitting parameters for the EIS spectra.

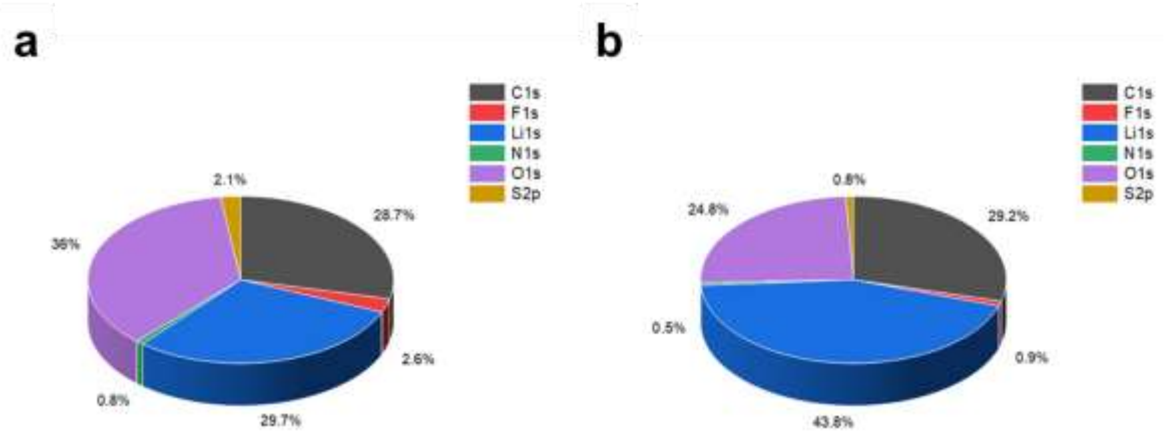

Supplementary Fig. 20 | Composition analysis based on XPS of the lithium metal anodes after 100 cycles, of fully discharged Li–S cells (i.e., 100% DOD) (**a**) Pristine PE and (**b**) TB-COF-TFSILi@PE.

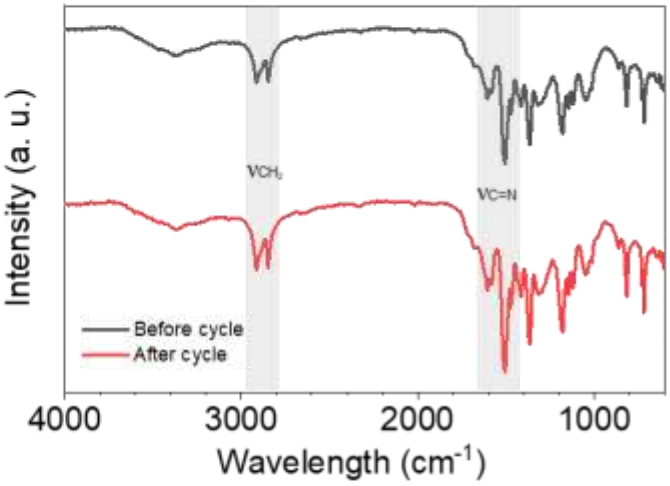

Supplementary Fig. 21 | FT-IR spectra of the TB-COF-TFSILi in the Li–S cells before and after 100 cycles.

Supplementary Table 1 | Estimated pore volume and pore size motif with total volume of functional groups in single COF framework. The pore diameter values were obtained by calculating the pore volume under the assumption of an ideal cylindrical shape.

COF membrane	***Number of COF layer***	***Volume of COF framework (□^{3})***	***Volume of functional motif (□^{3})***	***Estimated pore volume (□^{3})***	***Estimated pore size (nm)***	***Cross-sectional area of pore (□^{2})***
TB-COF-TFSILi			12674.8	4387.1	1.9	240.0
TB-COF-SO_3Li	6	17061.9	9139.9	7922.1	2.4	433.0
TB-COF			3459.9	13602.1	3.1	744.0

We investigated the effect of pore size on the flow of molecules using Darcy's law, an equation that simulates the flow of fluid through a porous medium:

$$Q = -\frac{kA}{\mu L}\Delta p$$

, where Q represents the volumetric flow rate; Δp is the pressure drop; μ is the fluid’s viscosity; k is the medium’s permeability; L is the distance through the porous structure; A is the cross-sectional area.

Given that the volumetric flow rate of molecules (Q) is directly proportional to the cross-sectional area (A), we calculated A through DFT calculations based on the pore diameters of TB-COF (3.1 nm), TB-COF-SO3Li (2.4 nm), and TB-COF-TFSILi (1.9 nm) as depicted in Supplementary Fig. 6. Our analysis revealed that the estimated pore diameters of six-layer stacked COFs correspond closely to the experimental results for the COFs examined herein. The corresponding cross-sectional area of TB-COF-TFSILi (239.96 Å^2) was the smallest compared to those of TB-COF-SO_3Li (433.0 Å^2) and TB-COF (744.0 Å^2). The decrease in the cross-sectional area in TB-COF-TFSILi could affect the volumetric flow rate of bulky PSs and $TFSI^-$, thereby maximizing the electrostatic permselective effect based on Darcy's law.

Supplementary Table 2 | Li^+ transference number (t_{Li^+}) of pristine PE and various COF@PEs measured by a potentiostatic polarization method.

	**Applied voltage(mV)**	**I_o (μA)**	**I_s (μA)**	**R_o (Ω)**	**R_s (Ω)**	**t_{Li^+}**
**Pristine PE**	10	45.64	19.78	78.81	134.4	0.38
**TB-COF@PE**	10	44.77	26.58	71.3	106.24	0.56
**TB-COF-SO_3Li@PE**	10	33.19	25.13	72.09	94.57	0.76
**TB-COF-TFSILi@PE**	10	49.01	41.97	60.15	71.96	0.87

Supplementary Table 3 | The Li^+ diffusion coefficients obtained with both PE and COF@PEs.

	Diffusion coefficient ($\times 10^{-8}$ cm^2 s^{-1})			
	**Pristine PE**	**TB-COF@PE**	**TB-COF-SO_3Li@PE**	**TB-COF-TFSILi@PE**
**R1**	3.42	3.38	4.48	6.30
**R2**	2.32	4.52	5.07	9.47
**O1**	18.4	19.6	24.6	38.7
**O2**	15.1	13.5	15.8	25.5

Supplementary Table 4 | Comparison of capacity decay rates of a discharge current density of 0.2 C between the Li–S cell with TB-COF-TFSILi@PE (this study) and previously reported Li–S cells.

Coating materials for separator membranes	Initial discharge capacity (mAh g^{-1})	Capacity retention (%)	Cycle number	Capacity decay rates	Capacity decay per cycle (%)	Average Coulombic efficiency (%)	Reference
**TB-COF-TFSILi**	**865**	**92.6%**	**250**	**7.4%**	**0.034%**	**98.1**	***This work***
**SPLTOPD**	1168	58%	210	32%	1.7 %	> 99.0	Ref. S5.
**TaVG-PP**	1302	83.2%	100	16.8%	0.085%	99.0	Ref. S6.
**Porous nitrogen-doped graphene**	977	84.5%	200	15.5%	0.075%	Not mentioned	Ref. S7.
**Co-N-C/rGO**	865	71.2%	500	28.8%	0.06%	98%	Ref. S8.
**SnO_2**	622	68%	500	32%	0.064%	Not mentioned	Ref. S9.
**N-doped-carbon nanowire**	1389	60%	200	40%	0.20%	98.0	Ref. S10.

**Carbon black**	1350	55%	500	45%	0.09%	95.0	Ref. S11.
**Microporous carbon/PEG**	1307	45%	500	55%	0.11%	Not mentioned	Ref. S12.
**MWNT/PEG**	1206	52%	300	0.48%	0.16%	Not mentioned	Ref. S13.
**SWNT**	1132	44.2%	300	55.8%	0.19%	Not mentioned	Ref. S14.
**Al_2O_3/Graphene**	1067.7	75%	100	25%	0.25%	Not mentioned	Ref. S15.
**Al_2O_3/CNT**	1287	63%	100	37%	0.37%	> 95.0	Ref. S16.
**Al_2O_3**	967	61.5%	50	38.5%	0.77%	99.0	Ref. S17.

Supplementary Table 5 | Comparison of Li–S cell with TB-COF-TFSILi@PE (this study) and previously reported Li–S cells, with a focus on capacity decay rate, cell capacity, and E/S ratio.

**References**	**Category**	**E/S ratio (μL mg_{sulfur}^{-1})**	**Cell capacity (mAh)**	**Capacity decay rate (%)**
This work	Membrane (COF)	4.5	8.3	0.046
Ref. 12	Sulfur cathodes	25	4.4	0.24
Ref. 13	Sulfur cathodes	18	9.8	0.042
Ref. 14	Sulfur cathodes	-	9.5	0.66
Ref. 15	Sulfur cathodes	16	12.2	0.68
Ref. 16	Sulfur cathodes	15	9.8	0.043
Ref. 17	Sulfur cathodes	-	1.3	0.11
Ref. 18	Electrolyte	3	3.1	0.047
Ref. 19	Electrolyte	6.3	8.1	0.83
Ref. 20	Protective layer	8	1.8	0.20
Ref. 21	Protective layer	-	1.4	0.20
Ref. 22	Membrane (carbon)	-	2.5	0.17
Ref. 23	Membrane (carbon)	60	3.1	0.024
Ref. 24	Membrane (carbon)	5	5.0	0.036
Ref. 25	Membrane (inorganic)	10	6.3	0.02

Ref. 26	Membrane (inorganic)	6.3	5.6	0.09
Ref. 27	Membrane (inorganic)	20	1.2	0.012
Ref. 28	Membrane (hybrid)	6.3	6.2	0.12
Ref. 29	Membrane (hybrid)	-	1.4	0.029
Ref. 55	Membrane (COF)	10	3.1	0.092
Ref. 57	Membrane (COF)	6	8.6	0.35
Ref. 58	Membrane (COF)	4	2.5	0.081
Ref. S18	Membrane (COF)	5	6.9	0.18
Ref. S19	Membrane (COF)	20	6.8	0.22
Ref. S20	Membrane (COF)	20	1.9	0.083
Ref. S21	Membrane (COF)	40	2.2	0.057
Ref. S22	Membrane (COF)	10	4.3	0.22
Ref. S23	Membrane (COF)	-	8.3	0.64

Supplementary Table 6 | Comparison of electrochemical properties of Li–S cells with various COF-based membranes.

References	**Membrane thickness (μm)**	**Mass Loading ($mg\ cm^{-2}$)**	**E/S ratio ($\mu L\ mg_{sulfur}^{-1}$)**	**Li foil (μm)**
This study	0.6 (COF)	8.3 (coin cell)	4.5	50
		5.2 (pouch cell)		
Ref. S19	9.23 (0.23 (COF) + 9 (CNT))	1.5 (coin cell)	~30	Not mentioned (estimated to be > 200 μm)
		5.4 (coin cell)		
Ref. S24	18	0.8 ~ 1 (coin cell)	20	Not mentioned (estimated to be > 200 μm)
		~ 9 (coin cell)	6	
Ref. S25	10 (CE-COF @50% CNT)	~1 (coin cell)	30 ~ 40	Not mentioned (estimated to be > 200 μm)
		8.04 (coin cell)	4	

References

S1. Zhi, Y. *et al*. Covalent organic frameworks as metal-free heterogeneous photocatalysts for organic transformations. *J Mater Chem A* 2017; **5**: 22933-22938.

S2. Xu, H. Tao, S. & Jiang, D. Proton conduction in crystalline and porous covalent organic frameworks. *Nat Mater* 2016; **15**: 722-726.

S3. Li, S. *et al*. Single-ion homopolymer electrolytes with high transference number prepared by click chemistry and photoinduced metal-free atom-transfer radical polymerization. *ACS Energy Lett* 2017; **3**: 20-27.

S4. Dimitrov, I. Jankova, k. & Hvilsted, S. Synthesis of polystyrene‐based random copolymers with balanced number of basic or acidic functional groups. *J Polym Sci Part A: Polym Chem* 2010; **48**: 2044-2052.

S5. Xia, S. *et al.* A separator with double coatings of $Li_4Ti_5O_{12}$ and conductive carbon for Li–S battery of good electrochemical perfor mance. *Adv. Sci.* **10**, 2301386 (2023).

S6. Zhao, Q. *et al.* Separator engineering toward practical Li–S batteries: Targeted electrocatalytic sulfur conversion, lithium plating regulation, and thermal tolerance. *Nano Energy* **95**, 106982 (2022).

S7. Xia, J. *et al.* Boosting catalytic activity by seeding nanocatalysts onto interlayers to inhibit polysulfide shuttling in Li–S batteries. *Adv. Funct. Mater.* **31**, 2101980 (2021).

S8. Chen, G. *et al.* A multifunctional separator modified with cobalt and nitrogen co-doped porous carbon nanofibers for Li–S batteries. *J. Membr. Sci.* **548**, 247–253 (2018).

S9. Xiang, Y. *et al.* Interfacing soluble polysulfides with a SnO_2 functionalized separator: An efficient approach for improving performance of Li–S battery. *J. Membr. Sci.* **563**, 380–387 (2018).

S10. Chung, S. H. & Manthiram, A. Bifunctional separator with a lightweight carbon-coating for dynamically and statically stable lithium–sulfur batteries. *Adv. Funct. Mater.* **24**, 5299–5306 (2014).

S11. Yao, H. *et al.* Improved lithium–sulfur batteries with a conductive coating on the separator to prevent the accumulation of inactive S-related species at the cathode–separator interface. *Energy Environ. Sci.* **7**, 3381–3390 (2014).

S12. Chung, S.-H. & Manthiram, A. A polyethylene glycol-supported microporous carbon coating as a polysulfide trap for utilizing pure sulfur cathodes in lithium–sulfur batteries. *Adv. Mater.* **26**, 7352–7357 (2014).

S13. Lu, Q. *et al.* An “electronegative” bifunctional coating layer: simultaneous regulation of polysulfide and Li-ion adsorption sites for long-cycling and dendrite-free Li–S batteries. *J. Mater. Chem. A* **7**, 22463–22474 (2019).

S14. Chang, C.-H., Chung, S.-H. & Manthiram, A. Effective stabilization of a high-loading sulfur cathode and a lithium-metal anode in Li–S batteries utilizing SWCNT-modulated separators. *Small* **12**, 174–179 (2015).

S15. Song, R. *et al.* A trilayer separator with dual function for high performance lithium–sulfur batteries. *J. Power Sources* **301**, 179–186 (2016).

S16. Xu, Q., Hu, G., Bi, H. & Xiang, H. A trilayer carbon nanotube/Al_2O_3 /polypropylene separator for lithium-sulfur batteries. *Ionics* **21**, 981–986 (2015).

S17. Zhang, Z. *et al.* Al_2O_3 -coated porous separator for enhanced electrochemical performance of lithium sulfur batteries. *Electrochim. Acta* **129**, 55–61 (2014).

S18. Xu, J. *et al.* Towards high-performance Li–S batteries via sulfonate‐rich COF‐modified separator. *Adv. Mater.* **33**, 2105178 (2021).

S19. Cao, Y. *et al.* Ion selective covalent organic framework enabling enhanced electrochemical performance of lithium–sulfur batteries. *Nano Lett.* **21**, 2997–3006 (2021).

S20. Wang, R. *et al.* An n-type benzobisthiadiazole-based covalent organic framework with narrowed bandgap and enhanced electroactivity. *Chem. Mater.* **33**, 3566–3574 (2021).

S21. Cao, Y. *et al.* Lithiation of covalent organic framework nanosheets facilitating lithium-ion transport in lithium-sulfur batteries. *Energy Storage Mater.* **29**, 207–215 (2020).

S22. Shi, Q. X. *et al.* Large-scaled covalent triazine framework modified separator as efficient inhibit polysulfide shuttling in Li–S batteries. *Chem. Eng. J.* **375**, 121977 (2019).

S23. Yan, W. *et al.* Boosting polysulfide catalytic conversion and facilitating Li^+ transportation by ion‐selective COFs composite nanowire for Li–S batteries. *Small* **18**, 2106679 (2022).

S24. Zhang, K. *et al.* Fluorinated covalent organic framework-based nanofluidic interface for robust lithium–sulfur batteries. *ACS Nano* **17**, 2901–2911 (2023).

S25. Han, D. *et al.* Supramolecular channels via crown ether functionalized covalent organic frameworks for boosting polysulfides conversion in Li–S batteries. *Energy Storage Mater.* **65**, 103143 (2024).
